# Event-Based Bidirectional People Counting Using a Spatiotemporal Neural Network and a Single 77 GHz MIMO FMCW Radar

**DOI:** 10.3390/s26196270

**Published:** 2026-10-03

**Authors:** Anna Ślesicka, Adam Kawalec, Błażej Ślesicki

**Affiliations:** 1 Department of Navigation, Faculty of Navigation and Logistics, Polish Air Force University, 08-521 Dęblin, Poland; 2 Faculty of Mechatronics, Armament, and Aerospace, Military University of Technology, 00-908 Warsaw, Poland; adam.kawalec@wat.edu.pl; 3 Department of Avionics and Control Systems, Faculty of Aviation Division, Polish Air Force University, 08-521 Dęblin, Poland; b.slesicki@law.mil.pl

**Keywords:** mmWave radar, FMCW radar, MIMO radar, bidirectional people counting, room occupancy estimation, radar point clouds, PointNet, BiGRU, attention mechanism

## Abstract

**Highlights:**

**What are the main findings?**

- The event-based model combining a PointNet encoder with a bidirectional gated recurrent unit network achieved a joint entry–exit count accuracy of 92.7% and a mean absolute error of 0.06 persons per event in estimating occupancy change. When applied without retraining or parameter adaptation in an independent laboratory, it retained a joint count accuracy of 91.0%.
- Geometric-consistency verification and the rejection of low-confidence decisions reduced cumulative counting errors, resulting in a mean absolute occupancy error of 0.10 person across five continuous sequences.

**What are the implications of the main findings?**

- By treating a complete doorway passage as a single event, the proposed method uses changes in point-cloud geometry, radial velocity, and their temporal evolution before, during, and after temporary merging of reflections. It therefore does not require continuous individual trajectories or frame-to-frame data association.
- Bidirectional people counting can be performed using standard radar point clouds generated by the device’s demonstration software, without using camera images as model inputs, accessing raw analog-to-digital converter samples, or modifying the radar firmware. The cross-environment experiment provides preliminary evidence of transferability, although further validation across additional rooms, doorway widths, participants, and radar mounting configurations is required.

**Abstract:**

Accurate bidirectional people counting at room entrances is essential for reliable occupancy estimation, yet radar-based methods remain challenged when reflections from multiple individuals temporarily merge or when people move simultaneously in opposite directions. This study presents a hybrid event-based method for bidirectional people counting using a single 77 GHz multiple-input multiple-output frequency-modulated continuous-wave radar. Unlike conventional tracking-based approaches, the proposed method treats a complete doorway passage as one event and does not require continuous individual trajectories or frame-to-frame data association. Each event is represented as a sequence of three-dimensional point clouds with radial velocity information. A PointNet encoder extracts frame-level spatial representations, a bidirectional gated recurrent unit models their temporal evolution, and an attention mechanism emphasizes the most informative event phases. Separate output heads estimate the numbers of entering and exiting individuals, while an auxiliary head identifies the event type. Predictions are accepted using explicit confidence and geometric-consistency criteria. The primary dataset comprised 550 labeled events and an additional 100 events recorded in five continuous sequences. In the held-out test session, entry- and exit-count accuracies were 95.5% and 94.5%, respectively, while joint entry–exit count accuracy reached 92.7%. The mean absolute error in occupancy change was 0.06 person per event, and the mean absolute occupancy error across the continuous sequences was 0.10 person. The proposed method exceeded geometric rules and density-based clustering combined with tracking in joint count accuracy by 37.2 and 24.5 percentage points, respectively. On an independent laboratory dataset comprising 100 events, the model achieved a joint count accuracy of 91.0% without retraining, fine-tuning, or parameter adaptation, providing preliminary evidence of cross-environment generalization.

## 1. Introduction

Reliable information on the number of people occupying a room is an important component of modern building management. It can be used to control ventilation, heating, and air-conditioning systems, as well as to manage lighting, assess space utilization, and support security systems [1]. Unlike simple presence detection, accurate people counting enables the operation of building systems to be adjusted to the actual occupancy of a room. This is particularly important in facilities where the number of occupants varies over time and does not follow fixed operating schedules. Studies on occupancy-based control of heating, ventilation, and air-conditioning (HVAC) systems confirm that information on the actual presence and number of occupants can help reduce energy consumption while maintaining the required indoor environmental quality [2]. In locations requiring enhanced supervision, automated security systems can be developed using information on the presence or density of people [3]. In commercial applications, information on the number of customers near individual service points can be useful both for identifying purchasing trends and for developing smart consumer services [4].

Occupancy monitoring technologies include passive infrared (PIR) sensors [5], optical barriers [6], RGB cameras [7], thermal cameras [8], carbon dioxide concentration sensors [9], and devices that monitor wireless network activity [10]. These solutions differ in the scope of information they provide, their accuracy, and their impact on user privacy. PIR sensors primarily detect movement and may fail to identify stationary occupants. CO_2_ concentration is an indirect indicator of occupancy, and its variation also depends on ventilation performance, room volume, and previous usage patterns. Vision-based systems provide rich spatial information; however, their effectiveness may be limited by changes in illumination and occlusions between people, while image acquisition introduces additional privacy-protection requirements.

Short-range radars operating in millimeter-wave frequency bands provide an alternative to optical sensors. Frequency-modulated continuous-wave (FMCW) radar enables the contactless estimation of range and radial velocity and, in a multiple-input multiple-output (MIMO) antenna configuration, the direction of arrival of the reflected signal [11]. Combining range, Doppler, and angular information provides a spatial representation of the observed scene in the form of a radar point cloud. Radar operation does not require illumination, and the acquired data do not contain direct images that would enable the visual identification of individuals. These properties make radar systems suitable for use in residential buildings, offices, and public facilities.

Previous applications of FMCW radar to people monitoring have included localization, tracking, classification, and counting. Knudde et al. used a low-power 77 GHz MIMO FMCW radar to track multiple people in an indoor environment [12]. The proposed processing pipeline comprised beamforming, background modeling, false-detection removal, and data association using Markov chain Monte Carlo data association (MCMCDA). The feasibility of detecting, tracking, and classifying multiple people using a compact FMCW radar was also demonstrated for a system operating at 24 GHz [13]. The robustness of radar-based tracking was also investigated in scenarios involving multiple users located near one another [14]. These studies did not directly count entries and exits but addressed the detection and tracking stages subsequently employed in people-counting systems.

In conventional bidirectional people-counting systems, the direction of passage is typically determined from the trajectory relative to predefined lines or boundary regions. Yang et al. developed a method using radar point clouds, multiscale clustering, and multi-object tracking of extended targets [15]. Following range, angle, and velocity estimation, microclustering, macroclustering, and refined clustering were applied sequentially, followed by two-stage data association. The numbers of entering and exiting people were determined from the resulting trajectories and the locations of the boundary regions.

A subsequent method accounted for interference caused by a moving door leaf [16]. Door-opening and door-closing events were detected using 4D radar data, after which points associated with the door leaf were extracted and clustered. The door was then incorporated into the tracking process as a separate target type. Different track-management rules were applied to doors and people, whereas the numbers of entries and exits were determined from the trajectories of the individuals and the locations of the boundary regions. Owing to the design of both algorithms, counting accuracy depends on the quality of point detection, clustering, data association, and the maintenance of valid trajectories for the tracked individuals.

Counting people moving in proximity as a single group is particularly challenging. If the distances between individuals are smaller than the radar’s range or azimuth resolution and their velocities are similar, it becomes difficult to distinguish individual people based on their positions and Doppler components. Ren et al. treated such a formation as a group target and combined a multi-target tracking algorithm with a classifier that estimated the number of people in each tracked group [17]. The classifier used features extracted from range–azimuth maps and cadence–velocity diagrams (CVDs), and the method was evaluated in outdoor scenarios. Wang et al. extended this approach to indoor environments by accounting for frequent changes in direction and stop-and-go motion [18]. They employed extended Kalman filter-based tracking and a classifier using spatial features extracted from range–azimuth maps and Doppler-frequency features obtained through wavelet decomposition. The processing output was a time-varying estimate of the group’s position and the number of people within it. Neither method requires a separate trajectory to be extracted for each person in the group; however, both still combine an explicit group-tracking module with a group-size classifier. Their outputs describe the current position and size of the observed group rather than the numbers of entries and exits or cumulative room occupancy.

In parallel, methods were developed in which the number of people was estimated from sequences of radar signals without prior detection and tracking of individual trajectories. Choi et al. developed Precent, whose input consisted of frames formed from consecutive preprocessed one-dimensional range profiles [19]. Features from each profile were extracted using a one-dimensional convolutional neural network (1D CNN), temporal dependencies were modeled using a bidirectional long short-term memory (BiLSTM) network, and the resulting representations were fused using an attention mechanism. The feature extractor was pretrained in an unsupervised manner as part of a one-dimensional convolutional autoencoder, after which the entire network was fine-tuned in a supervised manner for people-count regression. The method demonstrated the feasibility of automatically extracting features relevant to radar-based people counting. However, it used an impulse-radio ultra-wideband (IR-UWB) radar rather than an FMCW radar, and the model output represented the current number of people in the monitored area rather than an entry or exit event or the cumulative room occupancy.

For FMCW radar, Aydogdu et al. used a 60 GHz radar and a two-channel representation comprising two range–angle maps generated separately from the macro- and micro-Doppler components [20]. The radar convolutional neural network was trained both using radar data and through knowledge distillation from a vision-based network processing synchronized camera images. During inference, only radar data were used. The classifier distinguished classes corresponding to the presence of one, two, three, and four people, as well as a class representing more than four people. The method estimated the current number of people within the radar’s field of view but did not determine their direction of movement. Cao et al. presented a solution more closely related to counting people crossing an entrance [21]. The authors used range–time maps and a procedure for extracting the portion of the map corresponding to a passage event based on Doppler spectrum analysis. They subsequently applied a lightweight multiscale MFSNet within a joint-learning architecture comprising multiple encoders and a classifier. Using a single FMCW radar, the system simultaneously recognized the number of people passing through the monitored area and their direction of movement.

Deep learning has also been applied to representations and features derived from FMCW radar data acquired in the 24, 60, and 77 GHz frequency bands. Cakoni et al. classified group size using micro-Doppler signatures obtained from a 77 GHz FMCW radar [22]. The authors employed a custom CNN and compared its performance with existing image-classification networks applied through transfer learning. Martín-Martín et al. proposed an indoor people-counting system using a 60 GHz FMCW radar and a spiking neural network (SNN) that processed preprocessed range–Doppler maps [23]. Its performance was compared with that of the VGG16, ResNet-50, and MobileNet architectures in scenarios involving zero to five people. Wang et al. used a Transformer network to estimate the number of people in a tracked group from multi-frame sequences of spatial and frequency-domain features extracted from 24 GHz MIMO FMCW radar data [24]. The model was integrated into a tracking pipeline that enabled the simultaneous monitoring of the group’s position and size. Zhang et al., in turn, employed an improved ResNet-50 network and fused range–time and Doppler–time spectrograms obtained using a 60 GHz radar [25]. The system distinguished six composite classes describing the number of people and their motion configuration and was additionally evaluated in a separate task involving the counting of zero to three people.

The reviewed approaches address three types of tasks. The first group of methods counts people based on trajectories crossing a predefined boundary. The second estimates the number of people currently present in an observed scene or tracked group. The third comprises methods that simultaneously determine the number of people passing through the monitored area and their direction of movement from radar data [15,16,17,18,19,20,21,22,23,24,25]. However, to the best of the authors’ knowledge, the reviewed studies do not present a method that treats an entire passage through a doorway zone as a single event and analyzes it using sequences of three-dimensional MIMO FMCW radar point clouds. A solution is therefore still needed that can determine the number of entering and exiting people without continuously tracking each individual and subsequently update the current room occupancy.

This problem becomes particularly important when several people move in close proximity, and their reflections temporarily form a single cluster or extended object. Additional difficulties arise when people move simultaneously in opposite directions, stop within the doorway zone, change their speed, reverse direction before crossing the threshold, or pass by the entrance. Under such conditions, conventional tracking methods require correct data association and trajectory continuity, whereas classification of the current number of people in the scene does not unambiguously determine how many individuals crossed the threshold in each direction. Furthermore, even a small number of incorrect decisions for individual events may cause progressive drift between the estimated and actual occupancy.

Complete-event analysis reduces the dependence of the final result on the correct separation of individuals in every frame. The model can use information available before, during, and after the temporary merging of radar reflections and does not require persistent person identities or uninterrupted individual trajectories. The relevant information is contained in the evolution of the complete spatial point distribution and radial velocities between the entrance and inner zones rather than in the continuous assignment of each detection to a particular person.

The objective of this study is not merely to perform binary presence detection, which can be achieved using considerably simpler sensing solutions. Instead, the objective is to develop and evaluate a method that separately determines the numbers of entering and exiting people for each complete doorway event and subsequently updates the current room occupancy. Particular attention is given to challenging events in which several people move simultaneously, including in opposite directions, and their radar reflections temporarily merge, preventing the reliable maintenance of separate trajectories.

To achieve this objective, a hybrid event-based method was developed for bidirectional people counting and room occupancy estimation using a single 77 GHz MIMO FMCW radar. The unit of analysis is neither an individual frame nor the instantaneous number of people in the observed scene, but a complete event comprising the appearance of individuals within the doorway zone, their movement relative to the threshold, and their departure from the monitored area.

The input data consist of sequences of three-dimensional radar point clouds together with their corresponding radial velocities. These data are provided through the output stream of the radar demonstration software, referred to as the out-of-box (OOB) output. The method therefore uses data preprocessed by the radar and does not require access to raw analog-to-digital converter (ADC) samples or modification of the device firmware.

The core of the method is a multitask spatiotemporal neural network. A PointNet encoder processes the point cloud from each frame and produces a representation of its spatial distribution and radial velocities. A bidirectional gated recurrent unit (BiGRU) network then models the temporal evolution of successive representations, while the attention mechanism aggregates information from individual frames using learned weights. This enables the model to differentiate the contribution of successive event phases to the representation used for the final prediction. Spatial features describe the redistribution of radar activity between the entrance and inner zones and changes in the point-cloud geometry, whereas radial velocities provide information about the direction and dynamics of movement. The BiGRU captures the evolution of these features throughout the complete event. Consequently, temporary merging of reflections does not necessarily determine the final decision when the preceding and subsequent phases contain sufficient information to identify the event. The model independently estimates the numbers of entering (*n_in_*) and exiting (*n_out_*) individuals, thereby enabling the analysis of events involving simultaneous movement in both directions. The signed occupancy change for the *k*-th event was defined as *ΔN_k_* = *n_in,k_* − *n_out,k_*. An auxiliary five-class classification head identifies unidirectional movement, simultaneous movement in both directions, reversal, lateral passage past the entrance, and a stop followed by a return toward the doorway.

The neural network predictions were combined with geometric verification of movement across the doorway plane and an assessment of decision confidence. The occupancy state is updated only after the event has ended, the movement of radar activity between the two sides of the threshold has been confirmed, and the adopted confidence criterion has been satisfied. If the model output conflicts with the geometric information or its confidence is insufficient, the event is marked as ambiguous and recorded in the system log but does not automatically update the counter. This approach separates the interpretation of complex events, performed by the neural model, from the deterministic mechanisms used to verify decisions and maintain the room occupancy state.

The methodological contribution does not lie in introducing new standalone neural-network layers, but in formulating bidirectional counting as complete-event analysis, independently estimating entry and exit counts, and integrating spatiotemporal inference with deterministic geometric verification and occupancy-state updating. The main contributions of this study are as follows:

1. A formulation of bidirectional people counting as the analysis of a complete doorway-zone event, with direct and independent estimation of the numbers of entering and exiting individuals, rather than the classification of individual frames or the instantaneous number of people in the scene;
2. A multitask PointNet–BiGRU architecture with an attention mechanism that exploits temporal changes in the geometry and radial velocities of radar point clouds, including periods in which reflections from multiple individuals temporarily merge;
3. The inclusion of mixed, apparent, and ambiguous events involving simultaneous movement in both directions, reversal, lateral passage past the entrance, and stopping without crossing the doorway threshold;
4. The integration of neural model predictions with geometric verification of doorway crossing and confidence assessment, together with evaluation at the individual-event level, in terms of the long-term accumulation of occupancy-state errors, and in an independent environment without model retraining or fine-tuning;
5. The implementation of the complete processing pipeline using OOB data from a single 77 GHz MIMO FMCW radar, without access to raw ADC samples or modification of the device firmware.

The remainder of this article is organized as follows. Section 2 presents the materials and methods, including the radar system configuration, OOB data acquisition, event segmentation, model architecture, dataset, and evaluation metrics. Section 3 presents experimental results, comparisons with baseline methods, and ablation studies. Section 4 discusses the results and the limitations of the proposed method, while Section 5 provides the conclusions.

## 2. Materials and Methods

This section presents the radar system configuration, experimental environment, data acquisition protocol, and the proposed method for bidirectional people counting and room occupancy updating. It also describes the preparation and annotation of the dataset, the model training procedure, the adopted evaluation metrics, and the computational performance of the complete system.

### 2.1. Radar Hardware and OOB Data Configuration

**Figure 1:**
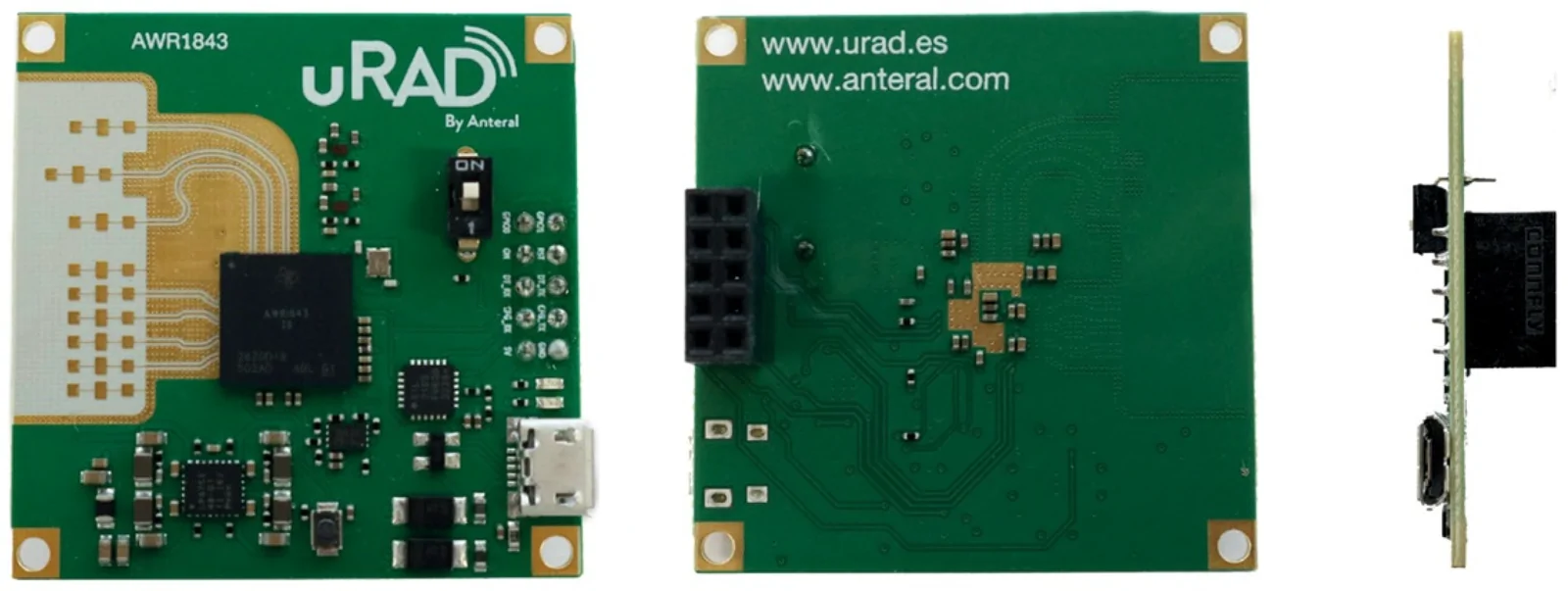
uRAD Automotive HPA v2.0 radar module based on the AWR1843 chipset used in the experiments: front, rear, and side views.

Measurement data were acquired using a uRAD Automotive HPA v2.0 radar (Anteral S.L., Huarte, Spain) equipped with a Texas Instruments AWR1843 device [26]. The radar board is shown in Figure 1. This compact FMCW radar operates over a frequency range of 76–81 GHz. Its antennas are integrated into the printed circuit board, which comprises three transmit and four receive antennas. In the configuration used in this study, all transmit and receive channels were activated, forming a MIMO array used to estimate the three-dimensional positions of the detected points. The field of view covers 120° in the azimuth plane and 40° in the elevation plane. The radar board measures 45 × 45 × 10 mm and weighs 5 g.

The FMCW waveform configuration was prepared using mmWave Demo Visualizer 3.6.0.0 for Texas Instruments mmWave SDK 3.06. The starting frequency was set to 77 GHz, and the chirp frequency slope was 70 MHz/µs. The chirp ramp time was 57.14 µs, while the idle time between consecutive chirps was 267 µs. A total of 256 complex samples were acquired during each chirp at a sampling rate of 5.209 MS/s. The total frequency sweep bandwidth during the chirp was approximately 4.00 GHz, whereas the effective bandwidth covered by the acquired samples was approximately 3.44 GHz. This corresponded to a range resolution of approximately 4.4 cm.

Transmission was performed using time-division multiplexing (TDM) of the transmit antennas. A single MIMO cycle comprised three consecutive chirps, each transmitted by a different antenna. The cycle was repeated 16 times, resulting in 48 chirps per frame. The frame period was 100 ms, corresponding to a frame rate of 10 frames per second. The maximum unambiguous range was approximately 9.02 m, while the detection range in the radar configuration was limited to 0–8.92 m. The maximum unambiguous radial velocity was ±1.00 m/s, with a velocity resolution of approximately 0.13 m/s.

Point detection was performed using a constant false alarm rate (CFAR) algorithm, with thresholds in the range and Doppler domains set to 15 dB. Peak grouping was enabled in both domains, together with multi-object beamforming. Hardware-based static clutter removal remained disabled, allowing static returns to be filtered during a subsequent software-processing stage. The principal parameters of the radar platform and the configuration used in this study are summarized in Table 1.

The radar was connected to the data acquisition computer through a micro-USB interface, which provided device power, configuration transfer, and output-data reception. The USB-to-UART bridge integrated into the radar provided two virtual serial ports. The configuration port operated at 115,200 bit/s and was used to transmit commands from the configuration file, whereas the data port operated at 921,600 bit/s and was used to receive successive measurement packets.

Data were acquired using the standard out-of-the-box firmware designed for radar point-cloud detection. The packet corresponding to each frame contained a header and type–length–value (TLV) data blocks. For each detection, the detected-points block contained the Cartesian coordinates together with the signed radial velocity. A supplementary side-information block contained the signal-to-noise ratio (SNR) and noise level associated with each point. The SNR and noise-level values were transmitted with a resolution of 0.1 dB. The number of points varied between frames and depended on the number of people within the field of view, their positions, movement patterns, and the strength of the generated reflections.

The coordinate-system origin was located at the center of the antenna array. From the perspective of an observer positioned behind the radar, the positive *X*-axis pointed to the right, the positive *Y*-axis pointed along the radar’s viewing direction, and the positive *Z*-axis pointed upward. The velocity provided by the device was the radial velocity, i.e., the component of the point velocity along the line connecting the point to the radar.

### 2.2. Experimental Environment, Participants, and Measurement Setup

**Figure 2:**
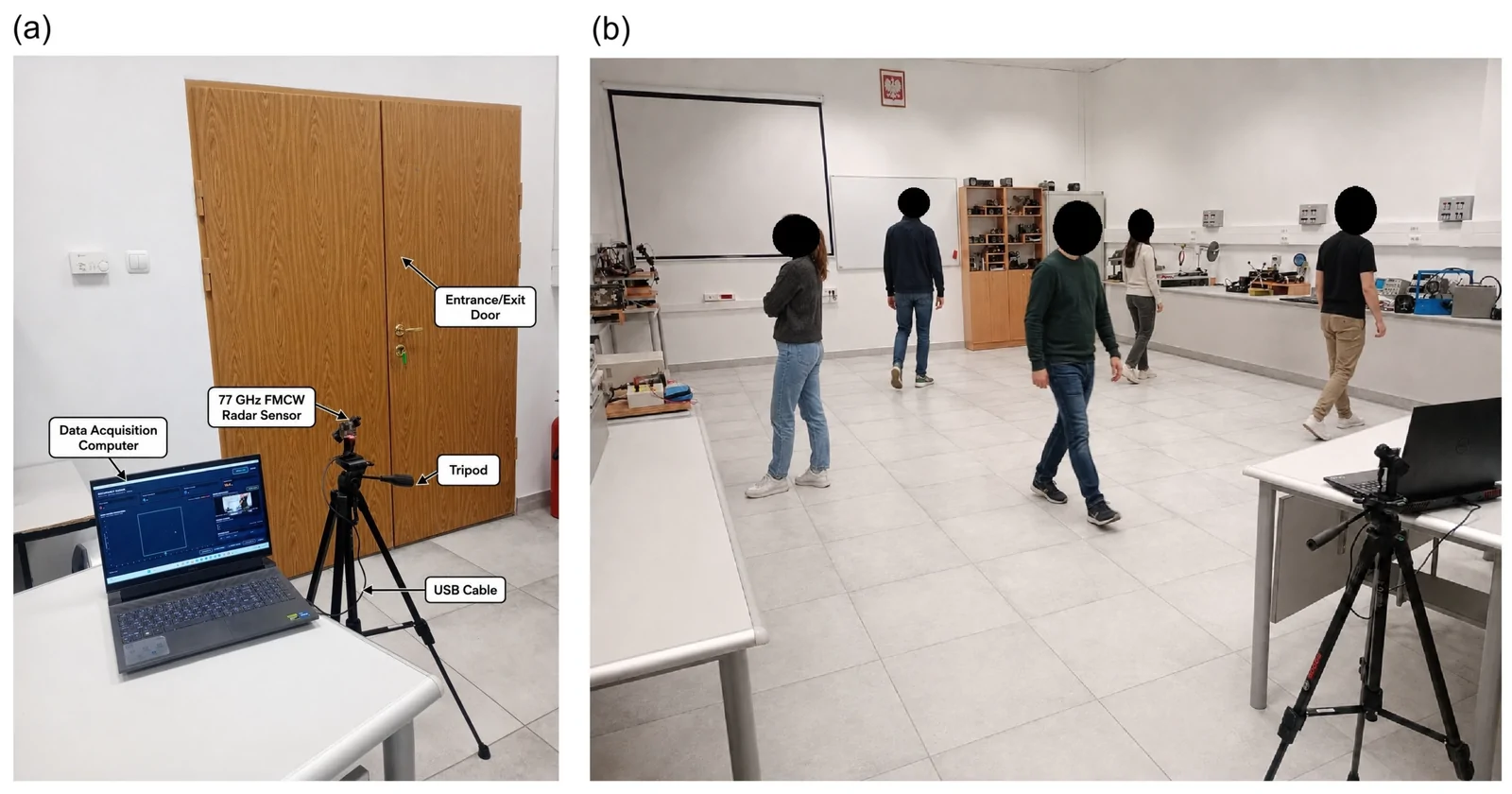
Experimental environment and measurement setup: (**a**) radar sensor and data-acquisition computer located inside the laboratory next to the entrance–exit door; (**b**) radar field of view directed toward the interior of the laboratory during multi-person measurements.

Measurements were conducted in a laboratory room measuring 5 m in width and 9 m in length, with a floor area of 45 m^2^. The laboratory contained typical furnishings and equipment, including tables, cabinets, measurement instruments, and computer hardware. These items were not removed during the experiment, allowing data to be acquired in an environment containing multipath reflections characteristic of real-world indoor spaces. An overall view of the laboratory environment is presented in Figure 2.

The measurement setup was located inside the laboratory, near the wall containing the entrance and exit door. The radar was positioned near the center of the room width, directly adjacent to the entrance zone. The sensor was mounted on a tripod at a height of 1 m above the floor and approximately 1 m from the doorway plane. The radar boresight was directed into the laboratory, approximately along its longitudinal axis. The sensor position and orientation remained unchanged throughout all measurement sessions.

During the measurements, the door remained fully open, and the position of its leaves did not change. This reduced variable radar reflections caused by the opening and closing of the door. The radar did not observe the area outside the room. Its field of view covered the entrance zone inside the laboratory and the space extending from the doorway toward the opposite end of the room.

The monitored area was divided into an entrance zone located directly adjacent to the radar and doorway and an inner zone covering the more distant part of the laboratory. When a person entered the room, radar activity first appeared in the entrance zone and subsequently moved farther into the laboratory. During an exit, movement in the opposite direction was observed: the activity moved from the inner zone toward the radar and disappeared near the doorway. The event direction was therefore determined from temporal changes in the position of the radar point cloud relative to the two zones, rather than through direct observation of both sides of the physical doorway plane. The measurement setup comprised the uRAD Automotive HPA v2.0 radar, a tripod, a USB cable, and a computer responsible for configuring the sensor, receiving the OOB stream, and recording the radar point clouds. The arrangement of the principal components of the setup is shown in Figure 2a.

A reference camera was used to record video simultaneously with radar data. The video recordings were used exclusively to establish the ground-truth course of each event, determine the number of people crossing the doorway threshold in each direction, and prepare the dataset labels. The camera images were not used as input to either the proposed method or the neural model.

Five university students aged 21–24 years participated in the experiment, including two women and three men. All participants were informed about the purpose and procedure of the experiment, the data acquisition process, and the intended use of the data in the study. Each participant provided informed consent to participate in the measurements and to have a reference video recorded.

The measurement protocol included both actual doorway crossings and events that should not change the occupancy state. The first group comprised passages involving one to five people moving in the same direction. Entry and exit events were recorded separately. Mixed events, during which participants moved simultaneously in opposite directions, were also investigated. All configurations involving a total of two to five participants were included. These comprised one person entering and one exiting; two entering and one exiting; one entering and two exiting; three entering and one exiting; two entering and two exiting; one entering and three exiting; four entering and one exiting; three entering and two exiting; two entering and three exiting; and one entering and four exiting.

Events that did not change the occupancy state included a participant appearing in the entrance zone and returning toward the doorway without moving into the inner zone, passing laterally through the entrance zone without continuing farther into the laboratory, and stopping near the entrance before leaving the monitored area along the same path. Actual entry and exit events involving changes in speed or brief stops were also recorded. In such cases, after stopping, the participant continued moving between the entrance and inner zones. This made it possible to distinguish a temporary stop during an actual entry or exit from an event that should not update the occupancy state.

The recorded dataset comprised 550 individual events used for model development and evaluation. Twenty repetitions were performed for each combination of people count and movement direction. For each of the three event types that did not change occupancy state, 50 repetitions were recorded. The dataset size represented a compromise between scenario diversity, the ability to evaluate individual configurations, and the logistical cost of conducting the experiment. The detailed structure of this part of the dataset is presented in Table 2.

The measurements were conducted in five separate sessions, each comprising 110 events. In each session, four repetitions were performed for every combination of people count and movement direction, together with ten repetitions of each event type that did not change the occupancy state. Between successive recordings, the group composition, participant order, spacing between individuals, and movement speed were varied.

The dataset of 550 individual events was partitioned at the level of complete measurement sessions. Three sessions comprising 330 events (60%) were used for model training, one session containing 110 events (20%) was used for validation, and the remaining session, also comprising 110 events (20%), was reserved for the final test evaluation. Events originating from the same session were not distributed across different dataset subsets.

In addition to the individual events, five continuous measurement sequences were recorded, each comprising 20 consecutive events. Each sequence began from a known, manually specified occupancy state. Between events, participants could remain stationary within the inner zone. Their presence was still included in the reference occupancy state, even when they did not generate stable detections in the current radar point cloud. The sequences were therefore used to evaluate the maintenance of an occupancy state derived from previous entries and exits rather than the direct estimation of the number of stationary people from an individual frame.

The additional 100 events contained in the continuous sequences were not used for model training or parameter selection. These data were used exclusively to evaluate occupancy-state updating and the accumulation of counting errors. The complete measurement dataset therefore comprised a total of 650 events.

### 2.3. Proposed Event-Based People-Counting Pipeline

**Figure 3:**
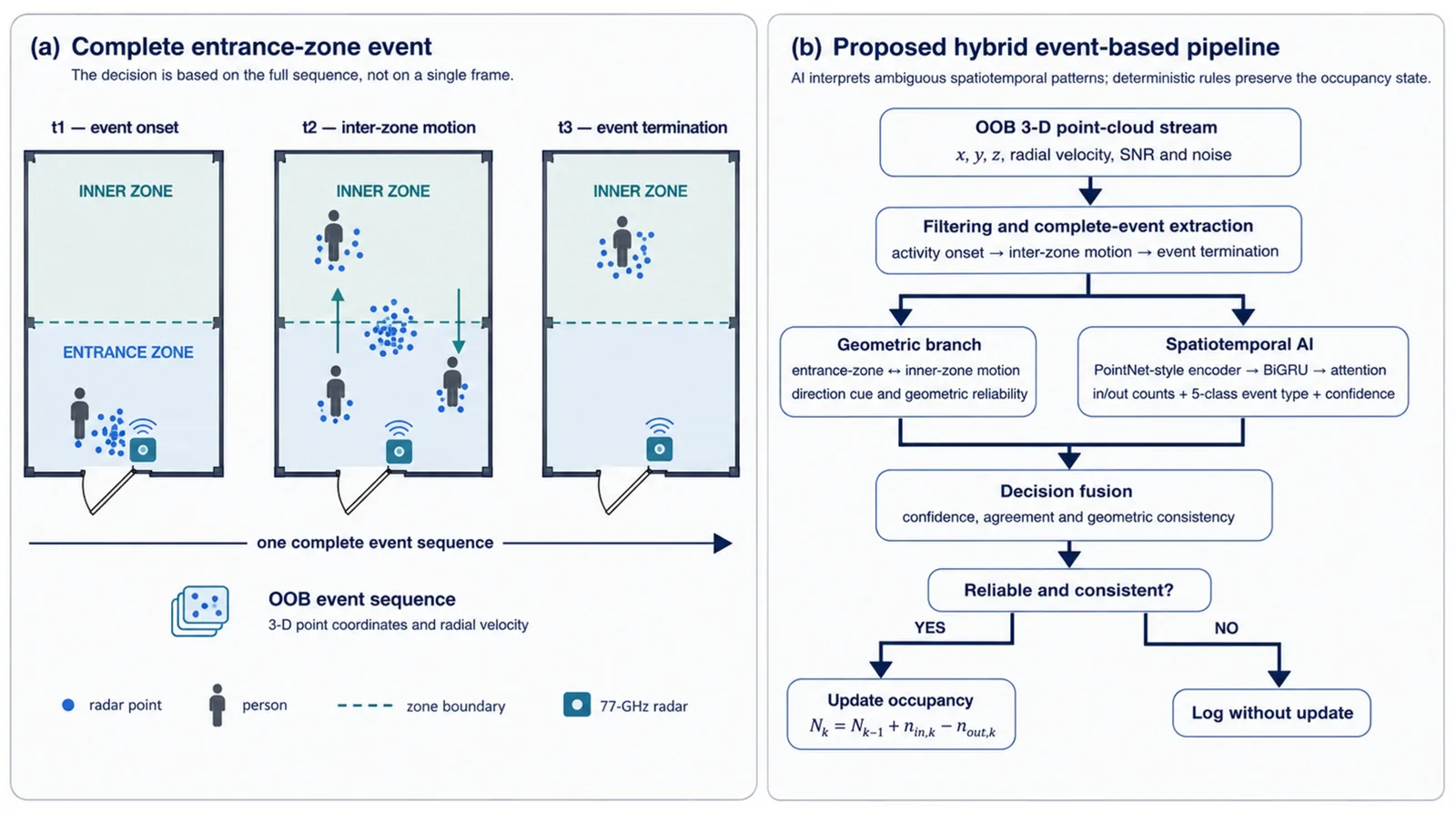
Overview of the proposed hybrid event-based people-counting pipeline: (**a**) complete entrance-zone event observed by a 77-GHz radar located inside the laboratory; (**b**) fusion of inter-zone geometric verification and spatiotemporal AI for bidirectional counting and occupancy-state updating.

The proposed method is a hybrid processing pipeline that combines event-based analysis of radar point clouds, a multitask spatiotemporal model, and a deterministic mechanism for maintaining the occupancy state. Following OOB data filtering and the extraction of a complete event, the resulting sequence is processed in parallel by a geometry-based branch and a PointNet–BiGRU model with an attention mechanism. The neural model estimates the numbers of people moving into the room and toward the exit, assigns the event to one of five classes, and determines the prediction confidence. The geometry-based branch evaluates the direction and consistency of radar-activity movement between the entrance and inner zones. The outputs of both branches are then combined, and the occupancy state is updated only for events that satisfy the adopted confidence and geometric-consistency criteria. The complete processing pipeline is illustrated in Figure 3.

The input consisted of a stream of OOB frames generated by the radar at a frequency of 10 Hz. The raw point cloud in frame *t* was expressed as

(1) $$P_{t}^{\mathrm{raw}}={\left\{p_{t,i}^{\mathrm{raw}}\right\}}_{i=1}^{M_{t}},$$

where *M_t_* denotes the number of detections in the frame, while an individual point was represented as

(2) $$p_{t,i}^{\mathrm{raw}}={\left[x_{t,i},y_{t,i},z_{t,i},v_{r,t,i},s_{t,i},\eta_{t,i}\right]}^{T}.$$

The quantities *x_t,i_*, *y_t,i_*, *z_t,i_* denote the spatial coordinates of the point relative to the radar; v*_r,t,i_* is its radial velocity; s*_t,i_* is the signal-to-noise ratio; and *η_t,i_* is the noise level. The coordinates and radial velocity were read from the TLV message containing the detected points, whereas the SNR and noise-level values were obtained from the corresponding side-information message.

In the first stage, points outside the measurement region, points with a very low absolute radial velocity, and detections with an insufficient SNR were discarded. The measurement region was defined as

(3) $$\Omega =\left[-4.0,4.0\right]\times \left[0.25,8.0\right]\times \left[-3.0,3.0\right]\;m.$$

The filtered point cloud was then determined according to the following relationship:

(4) $$P_{t}=\left\{p_{t,i}={\left[x_{t,i},y_{t,i},z_{t,i},v_{r,t,i}\right]}^{T}:\;\left(x_{t,i},y_{t,i},z_{t,i}\right)\in \Omega ,\;\left|v_{r,t,i}\right|\geq 0.05\;m/s,\;s_{t,i}\geq 5\;\mathrm{dB}\right\}.$$

The SNR and noise level were stored together with the complete measurement stream; however, the direct input to the neural model consisted of the spatial coordinates and radial velocities of the points satisfying the conditions specified above. Discarding points with very low radial velocities reduced the influence of reflections from stationary laboratory equipment.

The monitored region was divided using the boundary *y* = 2.0 m. The entrance zone was defined as *Z_E_* = {*p*: 0.25 ≤ *y* < 2.0 m}, whereas the inner zone was defined as *Z_I_* = {*p*: 2.0 ≤ *y* ≤ 8.0 m}. Points located exactly at *y* = 2.0 m were assigned to the inner zone. This convention was used consistently during event segmentation, geometric-consistency assessment, and occupancy-state updating. The numbers of active points in the two zones in frame *t* were defined as

(5) $$m_{E,t}=\left|P_{t}\cap Z_{E}\right|,\;\;m_{I,t}=\left|P_{t}\cap Z_{I}\right|.$$

A binary activity indicator was then defined as

(6) $$a_{t}=I\left(m_{E,t}+m_{I,t}\geq m_{\min}\right),$$

where *m_min_* denotes the minimum number of points required for a frame to be considered active. An event was initiated after activity was detected in *L_on_* consecutive frames and terminated after *L_off_* consecutive frames without activity. The start and end frame indices of the *k*-th event were defined as

(7) $$t_{s}^{\left(k\right)}=\min\left\{t:\overset{L_{\mathrm{on}}-1}{\underset{j=0}{\sum }}a_{t-j}=L_{\mathrm{on}}\right\},$$

(8) $$t_{e}^{\left(k\right)}=\min\left\{t>t_{s}^{\left(k\right)}:\overset{L_{\mathrm{off}}-1}{\underset{j=0}{\sum }}a_{t-j}=0\right\}.$$

The minimum activity threshold was set to *m_min_* = 3 points. Event onset was confirmed after *L_on_* = 3 consecutive active frames, whereas event termination was confirmed after *L_off_* = 10 consecutive inactive frames. At the radar frame rate of 10 Hz, these settings corresponded to onset and termination confirmation intervals of 0.3 s and 1.0 s, respectively. The longer termination interval reduced the risk of dividing a single passage into several shorter events when the number of detections temporarily decreased or a participant briefly stopped. These values were selected using the validation set and were subsequently fixed for all test experiments.

The complete *k*-th event was represented as an ordered sequence of filtered point clouds:

(9) $$E_{k}=\left(P_{t_{s}^{\left(k\right)}},P_{t_{s}^{\left(k\right)}+1},\ldots ,P_{t_{e}^{\left(k\right)}}\right).$$

Simple geometric rules are sufficient to determine the general direction of movement of a clearly separated target, but they cannot reliably determine the number of people involved in a complex event. The number of radar points is not directly proportional to the number of people because it depends on factors including body orientation, distance from the radar, mutual occlusion between participants, and received signal strength. Several people moving in proximity may temporarily form a single cluster, whereas reflections from one person may be divided into several disconnected clusters. Furthermore, stopping, reversing direction, and passing laterally through the entrance zone may resemble the initial phase of an actual entry or exit in individual frames. Therefore, the model input was not an individual frame but the complete event *E_k_*.

The model performed three related classification tasks. Two counting heads generated the probability vectors:

(10) $$p_{k}^{\mathrm{in}},p_{k}^{\mathrm{out}}\in {\left[0,1\right]}^{6},$$

corresponding to counts from zero to five people moving from the entrance zone to the inner zone and from the inner zone toward the exit, respectively. The count predictions were determined as

(11) $${\hat{n}}_{\mathrm{in},k}=\underset{j\in \left\{0,\ldots ,5\right\}}{\mathrm{argmax}}\;p_{k,j}^{\mathrm{in}},$$

(12) $${\hat{n}}_{out,k}=\underset{j\in \left\{0,\ldots ,5\right\}}{\mathrm{argmax}}\;p_{k,j}^{out},$$

The third head generated the vector:

(13) $$p_{k}^{\mathrm{event}}\in {\left[0,1\right]}^{5}$$

and assigned the event to one of five classes: unidirectional movement, simultaneous movement in opposite directions, reversal within the entrance zone, lateral passage through the entrance zone, or a stop followed by a return toward the doorway. The predicted event class was determined as

(14) $${\hat{c}}_{k}=\underset{r\in \left\{1,\ldots ,5\right\}}{\mathrm{argmax}}\;p_{k,r}^{\mathrm{event}}.$$

The separate estimation of ${\hat{n}}_{\mathrm{in},k}$ and ${\hat{n}}_{\mathrm{out},k}$ enabled the processing of mixed events for which both values were greater than zero.

The model outputs were subsequently subjected to a geometric consistency assessment. For each frame, the normalized balance of activity between the two zones was calculated as:

(15) $$b_{t}=\frac{m_{I,t}-m_{E,t}}{m_{I,t}+m_{E,t}+\varepsilon },$$

where *ε* prevents division by zero. The change in the position of radar activity during the *k*-th event was determined from the difference between the mean values of *b_t_* in the final and initial segments of the sequence:

(16) $$d_{k}=\frac{1}{W_{g}}\overset{t_{e}^{\left(k\right)}}{\underset{t=t_{e}^{\left(k\right)}-W_{g}+1}{\sum }}b_{t}-\frac{1}{W_{g}}\overset{t_{s}^{\left(k\right)}+W_{g}-1}{\underset{t=t_{s}^{\left(k\right)}}{\sum }}b_{t},$$

where *W_g_* denotes the number of frames used in each segment.

The geometric-consistency indicator was determined using fixed rules defined before the final test evaluation. The window length used to compare the beginning and end of an event was *W_g_* = 5 frames. Thus, *d_k_* represented the difference between the mean zone-balance value over the last five frames and that over the first five frames of the event.

For an entry-only prediction, defined by *n_in,k_* > 0 and *n_out,k_* = 0, geometric consistency was accepted when *d_k_* ≥ 0.15. For an exit-only prediction, defined by *n_out,k_* > 0 and *n_in,k_* = 0, consistency was accepted when *d_k_* ≤ −0.15.

Mixed events required a separate rule because simultaneous entry and exit may produce only a small net change in the zone-balance indicator. For this purpose, the fractions of event frames containing at least one point in the entrance and inner zones were calculated as

(17) $$r_{E,k}=\frac{1}{T_{k}}\overset{T_{k}}{\underset{t=1}{\sum }}I\left(m_{E,t}\geq 1\right)$$

(18) $$r_{I,k}=\frac{1}{T_{k}}\overset{T_{k}}{\underset{t=1}{\sum }}I\left(m_{I,t}\geq 1\right).$$

The temporal span of the zone-balance indicator was additionally calculated as

(19) $$s_{k}=\underset{1\leq t\leq T_{k}}{\max}\left(b_{t}\right)-\underset{1\leq t\leq T_{k}}{\min}\left(b_{t}\right).$$

A mixed event was considered geometrically consistent when *n_in,k_* > 0, *n_out,k_* > 0, the auxiliary event head predicted simultaneous movement in both directions, r*_E,k_* ≥ 0.15, r*_I,k_* ≥ 0.15, and *s_k_* ≥ 0.25. These conditions required measurable activity on both sides of the boundary and a sufficient change in its spatial distribution during the event.

For no-change events, including reversal, lateral passage, and stopping followed by a return toward the doorway, consistency was accepted when *n_in,k_* = 0, *n_out,k_* = 0, and |*d_k_*| ≤ 0.20. All other combinations of count predictions, event type, and geometric conditions were classified as geometrically inconsistent. Accordingly, *g_k_* was set to 1 when the relevant rule was satisfied and to 0 otherwise.

The prediction confidence for the complete event was defined as the minimum of the maximum probability values produced by the three heads:

(20) $$q_{k}=\min\left\{\underset{j}{\max}p_{k,j}^{\mathrm{in}},\underset{j}{\max}p_{k,j}^{\mathrm{out}},\underset{r}{\max}p_{k,r}^{\mathrm{event}}\right\}.$$

The confidence threshold was fixed at *τ_q_* = 0.80 based on the validation set and was not modified during final testing. A decision was accepted for occupancy-state updating if it satisfied the confidence threshold *τ_q_* and the geometric-consistency criterion:

(21) $$A_{k}=I\left(q_{k}\geq \tau_{q}\right)g_{k}.$$

Consequently, an event was allowed to update the occupancy state only when its minimum confidence across the three output heads was at least 0.80 and the corresponding geometric-consistency rule was satisfied.

For an accepted event, the occupancy change was calculated as

(22) $$\Delta {\hat{N}}_{k}={\hat{n}}_{\mathrm{in},k}-{\hat{n}}_{\mathrm{out},k}.$$

The occupancy state after the *k*-th event was updated according to

(23) $${\hat{N}}_{k}=\left\{\begin{array}{ll} \max\left(0,{\hat{N}}_{k-1}+\Delta {\hat{N}}_{k}\right), & A_{k}=1,\\ {\hat{N}}_{k-1}, & A_{k}=0. \end{array}\right.$$

Events that failed either the confidence criterion or the geometric-consistency condition were marked as ambiguous and recorded in the system log but did not automatically update the counter.

According to Equation (23), after the *k*-th event was accepted, the occupancy state was increased by the number of recognized entries and decreased by the number of recognized exits. The estimated state was retained between successive events; therefore, the method did not require continuous detection of every person present in the laboratory. A participant whose entry had previously been recognized remained included in the count even after stopping and no longer generating points that satisfied the filtering criteria. The participant was removed from the occupancy state only after an accepted event corresponding to their exit. The method did not directly estimate the number of stationary people from an individual frame. Because the counter represented occupancy changes resulting from successive events, its initial value ${\hat{N}}_{0}$ had to be known and set before the system was started. In the experiments, ${\hat{N}}_{0}$ = 0 was assumed.

In the proposed approach, deterministic rules were responsible for spatial and quality-based filtering of the point clouds, determining the temporal boundaries of events, assessing the consistency of movement direction between the entrance and inner zones, and maintaining the occupancy state. The neural model was used to estimate the numbers of people moving in both directions and to recognize the type of complex event from the complete sequence of spatiotemporal changes. This separation restricted the use of the neural model to the part of the problem for which the number of points, the number of clusters, and simple threshold-based and geometric relationships did not provide an unambiguous interpretation.

The model input was a sequence of radar point clouds corresponding to the complete event *E_k_*. Each filtered point was represented by a four-dimensional feature vector:

(24) $$u_{k,t,i}={\left[x_{k,t,i,}y_{k,t,i,}z_{k,t,i,}v_{k,t,i,}\right]}^{T}.$$

comprising three spatial coordinates and the radial velocity. The index *k* denotes the event number, *t* denotes the frame number within the sequence, and *i* denotes the point number within a given frame. The signal-to-noise ratio was used during the preceding processing stage as a detection-quality criterion but was not included as a separate input feature of the neural model. The model therefore analyzed the geometry of the point clouds and changes in radial velocity, whereas the SNR influenced only the selection of points passed to the subsequent processing stages.

Before the data were provided to the model, each of the four features was standardized according to the following relationship:

(25) $${\tilde{f}}_{k,t,i}=\frac{\left[f_{k,t,i}-\mu \left(f\right)\right]}{\sigma \left(f\right)},\;\;\;\;f\epsilon \left\{x,y,z,v_{r}\right\}$$

where *μ(f)* and *σ(f)* denote the mean and standard deviation of a given feature, respectively, calculated exclusively from the training set. The normalization parameters were kept unchanged when processing the validation and test sets.

Because the numbers of frames and points varied between events, each event was transformed into a tensor:

(26) $$X_{k}\epsilon {\mathbb{R}}^{\left(T_{max}\times P_{max}\times F\right)}={\mathbb{R}}^{\left(80\times 64\times 4\right)},$$

where *T_max_* = 80 is the maximum number of event frames, *P_max_* = 64 is the maximum number of points in a single frame, and *F* = 4 is the number of features describing each point. At a frame rate of 10 Hz, a sequence of 80 frames corresponded to an observation period of 8 s.

If a frame contained more than 64 points, 64 points were selected using farthest point sampling (FPS), which preserved the spatial extent of the point cloud. Frames containing fewer than 64 points were padded with zero vectors, and the padded positions were identified using a point mask. Events shorter than 80 frames were padded with empty frames at the end of the sequence, and a corresponding temporal mask was created. If an event contained more than 80 frames, 80 frames distributed uniformly over its entire duration were selected, thereby preserving the initial, middle, and final phases of the event. The point and temporal masks were used during feature aggregation and by the attention mechanism to prevent the artificially added elements from affecting the model output.

The first component of the network was a lightweight PointNet encoder applied independently to each frame of an event. The encoder comprised three shared fully connected layers that transformed the four-dimensional input vectors successively into feature representations of 32, 64, and 96 dimensions. The operation of the *l*-th encoder layer can be expressed as

(27) $$h_{k,t,i}^{\left(l\right)}=\mathrm{ReLU}\;\left[{\mathrm{BN}}_{l}\;\left(W_{l}h_{k,t,i}^{\left(l-1\right)}+b_{l}\right)\right],\;\;l\in \left\{1,2,3\right\}.$$

where h_k_,_t_,_i_^(0)^ = ũ_k_,_t_,_i_ denotes the normalized feature vector of the *i*-th point in frame *t* of event *k*, while *BN_l_* denotes batch normalization. The dimensions of the representations *h*^(1)^, *h*^(2)^ i *h*^(3)^ were 32, 64 and 96, respectively. The same weight matrices *W_l_* and bias vectors *b_l_* were applied to all points. Artificially added padding points were excluded from the calculations using the point mask.

After processing the individual points, masked global max pooling was performed. For the *j*-th feature, the frame representation was calculated as

(28) $$c_{k,t,j}=\underset{i\in V_{k,t}}{\max}h_{k,t,i,j}^{\left(3\right)},\;\;j\in \left\{1,\ldots ,96\right\},$$

where the maximum was calculated exclusively over the valid points identified by the mask. As a result of this operation, a point cloud containing up to 64 points was replaced by a 96-dimensional vector *c_k_*,*_t_*. Global max pooling made the frame representation independent of the order of the points. The vector *c_k_*,*_t_*. described the spatial distribution of the reflections and their radial velocities without requiring points to be assigned to individual people or their trajectories to be maintained.

The sequence of frame-level vectors was then passed to a single-layer bidirectional GRU network. A total of 64 hidden units were used in each direction. The hidden states were calculated according to the following relationships:

(29) $$h_{k,t}^{\left(f\right)}={\mathrm{GRU}}_{f}\;\left(c_{k,t},h_{k,t-1}^{\left(f\right)}\right),$$

(30) $$h_{k,t}^{\left(b\right)}={\mathrm{GRU}}_{b}\;\left(c_{k,t},h_{k,t+1}^{\left(b\right)}\right).$$

where *f* denotes the forward direction and *b* denotes the backward direction. The outputs from both directions were concatenated to obtain the following vector for each frame:

(31) $$h_{k,t}=\left[h_{k,t}^{\left(f\right)};h_{k,t}^{\left(b\right)}\right]\in {\mathbb{R}}^{128}.$$

The forward direction analyzed the evolution of the event from its beginning, whereas the backward direction incorporated information available from the end of the sequence. Bidirectional processing was possible because the counting decision was made only after the complete event had ended. Frames added to pad the sequence to a length of 80 were excluded using the temporal mask.

An additive attention mechanism was applied to the BiGRU outputs. A scalar attention score was calculated for each valid frame as

(32) $$e_{k,t}=w_{a}^{T}\tan h\;\left(W_{a}h_{k,t}+b_{a}\right).$$

The linear transformation defined by ***W***_*a*_ reduced the representation dimension from 128 to 64 features, whereas the vector ***W***_*a*_ produced a single scalar attention score for each valid frame. These values were normalized over time:

(33) $$\alpha_{k,t}=\frac{\exp\left(e_{k,t}\right)}{\sum_{j\in T_{k}}\exp\left(e_{k,j}\right)},$$

where the summation was performed exclusively over the valid frames of the event. The representation of the complete event was calculated as a weighted sum:

(34) $$z_{k}=\underset{t\in T_{k}}{\sum }\alpha_{k,t}h_{k,t},\;\;z_{k}\in {\mathbb{R}}^{128},$$

This produced a weighted representation of the complete event in which individual frames could contribute differently to the final prediction.

The roles of the input features and network components can be interpreted in relation to the successive phases of a doorway event. The spatial coordinates x, y, and z describe changes in the geometry and location of the point-cloud distribution, including the redistribution of activity between the entrance and inner zones. Radial velocity provides information about the direction and dynamics of movement relative to the radar. PointNet generates a frame-level representation without requiring point-to-point correspondence or the assignment of detections to individual people. The BiGRU then models the evolution of these representations before, during, and after the temporary merging of reflections. The attention mechanism produces a weighted event-level representation from the complete sequence. Therefore, the final prediction does not require continuous individual trajectories or persistent person identities and can use information available before and after a temporary loss of separability.

The representation ***z****_k_* was passed to a shared fully connected layer that reduced its dimensionality from 128 to 64. This layer was followed by a ReLU activation function and dropout with a rate of 0.3. The resulting 64-dimensional vector ***r****_k_* was provided as input to three independent classification heads. Each head contained an additional fully connected layer with 32 neurons followed by a ReLU activation function.

The first head generated six probabilities corresponding to counts from zero to five people moving from the entrance zone to the inner zone. The second head had an analogous structure and recognized the number of people moving from the inner zone toward the exit. The third head generated five probabilities corresponding to the following event classes:

1. Unidirectional movement between the two zones;
2. Simultaneous movement of people in opposite directions;
3. Reversal within the entrance zone;
4. Lateral passage through the entrance zone;
5. A stop within the entrance zone followed by a return toward the doorway.

The output vectors of the individual heads therefore had the following forms:

(35) $$p_{k}^{\mathrm{in}}\in {\left[0,1\right]}^{6},\;\;p_{k}^{\mathrm{out}}\in {\left[0,1\right]}^{6},\;\;p_{k}^{\mathrm{event}}\in {\left[0,1\right]}^{5}.$$

A softmax function was applied at the output of each head. The number of people and the event type were determined as the index of the class with the highest probability. The maximum probabilities produced by the three heads were used to calculate the confidence score *q_k_* for the complete event. The acceptance threshold *τ_q_* was selected using the validation set and was kept unchanged during test-set evaluation.

The proposed architecture contained a total of 94,482 trainable parameters. The model was trained from scratch without using weights from previously trained networks.

The model was trained in a multitask manner. The total loss function was defined as the sum of the losses from the three heads:

(36) $$L=\lambda_{\mathrm{in}}L_{\mathrm{in}}+\lambda_{\mathrm{out}}L_{\mathrm{out}}+\lambda_{\mathrm{event}}L_{\mathrm{event}},$$

where *L_in_*, *L_out_*, and *L_event_* denote the weighted cross-entropy losses for the entry count, exit count, and event type, respectively. Equal importance was assigned to all three tasks by setting:

(37) $$\lambda_{\mathrm{in}}=\lambda_{\mathrm{out}}=\lambda_{\mathrm{event}}=1.$$

For each head, the loss was defined as

(38) $$L_{s}=-\overset{C_{s}}{\underset{c=1}{\sum }}w_{s,c}\;y_{s,c}\ln\;\left(p_{s,c}\right),\;\;s\in \left\{\mathrm{in},\mathrm{out},\mathrm{event}\right\}.$$

where *s* denotes the task under consideration; *y_s,c_* is the ground-truth label for class *c*; *p_s,c_* is the probability predicted by the model; and *w_s,c_* is the weight assigned to that class. The class weights were calculated from the class frequencies in the training set to reduce the effect of the uneven distribution of people-count values and event types.

The AdamW optimizer was used for training. The initial learning rate was 0.001, the weight-decay coefficient was 0.0001, and the batch size was 16 events. The maximum number of training epochs was set to 150. Training was stopped if the total validation loss did not improve for 15 consecutive epochs. The gradient norm was clipped to 1.0 during each parameter update. The model parameters obtained in the epoch with the lowest validation loss were retained for subsequent evaluation.

Data augmentation during training included independent Gaussian perturbations of the spatial coordinates with a standard deviation of 0.01 m, perturbations of the radial velocity with a standard deviation of 0.02 m/s, and the random removal of up to 10% of the points from each frame. With a probability of 0.5, the point cloud was also mirrored laterally within the laboratory, corresponding to a sign change in the transverse coordinate without changing the direction of movement between the two zones. The frame order was not reversed because this operation would change the temporal direction of the event and would require the entry- and exit-count labels to be swapped.

### 2.4. Metrics

The final evaluation was conducted using 110 events from the test session, which was not used for model training, hyperparameter selection, or confidence-threshold determination. First, the raw predictions of the three model heads were evaluated for all test events. The complete hybrid method, including geometric verification and the rejection of events that did not satisfy the confidence criterion, was then evaluated.

Let M denote the number of test events, while $y_{s,k}$ and ${\hat{y}}_{s,k}$ denote the ground-truth and predicted classes, respectively, for the *k*-th event and task *s*. Three tasks were considered: estimation of the number of people moving into the laboratory (*s* = in), estimation of the number of people moving toward the exit (*s* = out), and event-type classification (*s* = type). The first two heads distinguished six classes corresponding to counts from zero to five people, whereas the event-type head distinguished five classes.

The primary performance measure for each head was classification accuracy:

(39) $${\mathrm{Acc}}_{s}=\frac{1}{M}\overset{M}{\underset{k=1}{\sum }}I\;\left({\hat{y}}_{s,k}=y_{s,k}\right),$$

where I(⋅) denotes an indicator function that takes a value of 1 when the specified condition is satisfied and 0 otherwise.

Owing to class imbalance, the macro-averaged *F1*-score was also calculated. For a class belonging to task *s*, *precision*, *recall*, and the *F1*-score were defined as

(40) $${\mathrm{Precision}}_{s,c}=\frac{{\mathrm{TP}}_{s,c}}{{\mathrm{TP}}_{s,c}+{\mathrm{FP}}_{s,c}},$$

(41) $${\mathrm{Recall}}_{s,c}=\frac{{\mathrm{TP}}_{s,c}}{{\mathrm{TP}}_{s,c}+{\mathrm{FN}}_{s,c}},$$

(42) $$F1_{s,c}=\frac{2\;{\mathrm{Precision}}_{s,c}{\mathrm{Recall}}_{s,c}}{{\mathrm{Precision}}_{s,c}+{\mathrm{Recall}}_{s,c}},$$

where *TP*, *FP*, and *FN* denote the numbers of true-positive, false-positive, and false-negative results, respectively. The macro-averaged *F1*-score was calculated as

(43) $$\mathrm{Macro}-F1_{s}=\frac{1}{C_{s}}\overset{C_{s}}{\underset{c=1}{\sum }}F1_{s,c},$$

where *C_s_* denotes the number of classes for a given task. The adopted values were *C_in_* = *C_out_* = 6 and *C_type_* = 5. Macro-averaging ensured that each class contributed equally to the result, regardless of the number of test examples belonging to that class.

Because the people count is an ordered variable, mean absolute error (*MAE*) was also calculated for both counting heads. For the numbers of people moving into the laboratory and toward the exit, it was defined as

(44) $${\mathrm{MAE}}_{\mathrm{in}}=\frac{1}{M}\overset{M}{\underset{k=1}{\sum }}\left|{\hat{n}}_{\mathrm{in},k}-n_{\mathrm{in},k}\right|,$$

(45) $${\mathrm{MAE}}_{\mathrm{out}}=\frac{1}{M}\overset{M}{\underset{k=1}{\sum }}\left|{\hat{n}}_{\mathrm{out},k}-n_{\mathrm{out},k}\right|,$$

Unlike classification accuracy, these metrics account for the magnitude of the error, distinguishing an error of one person from an error of several people.

To assess the simultaneous correctness of both counting heads, joint count accuracy (*JCA*) was calculated as

(46) $$\mathrm{JCA}=\frac{1}{M}\overset{M}{\underset{k=1}{\sum }}I\;\left({\hat{n}}_{\mathrm{in},k}=n_{\mathrm{in},k}\;\wedge \;{\hat{n}}_{\mathrm{out},k}=n_{\mathrm{out},k}\right),$$

Under this metric, an event was considered correctly recognized only when the numbers of people moving in both directions were simultaneously estimated correctly. This measure was particularly important for mixed events, for which a correct value from only one of the two heads did not provide a complete description of the people flow.

For each event, the ground-truth and predicted occupancy changes were calculated as:

(47) $$\Delta N_{k}=n_{\mathrm{in},k}-n_{\mathrm{out},k},$$

(48) $${\hat{\Delta N}}_{k}={\hat{n}}_{\mathrm{in},k}-{\hat{n}}_{\mathrm{out},k}.$$

The error in the signed occupancy change was quantified using the mean absolute error:

(49) $${\mathrm{MAE}}_{\Delta N}=\frac{1}{M}\overset{M}{\underset{k=1}{\sum }}\left|{\hat{\Delta N}}_{k}-\Delta N_{k}\right|.$$

This metric directly evaluated the occupancy-change value passed to the occupancy-state update mechanism. It was analyzed together with joint count accuracy because identical values of *ΔN* may result from different combinations of entry and exit counts.

The effects of confidence assessment and geometric verification were evaluated using coverage and selective accuracy. Let *A_k_* take a value of 1 if the *k*-th event was accepted for automatic counter updating and 0 if it was marked as ambiguous. *Coverage* was defined as

(50) $$\mathrm{Coverage}=\frac{1}{M}\overset{M}{\underset{k=1}{\sum }}A_{k}.$$

This value represented the proportion of events processed automatically by the system. *Selective accuracy* quantified the proportion of correctly estimated occupancy changes among the accepted events:

(51) $$\mathrm{Selective}\_\mathrm{Accuracy}=\frac{\sum_{k=1}^{M}A_{k}\;I\;\left({\hat{\Delta N}}_{k}=\Delta N_{k}\right)}{\sum_{k=1}^{M}A_{k}}.$$

Reporting coverage together with selective accuracy prevented an apparent improvement in accuracy resulting solely from the rejection of a large proportion of difficult events.

The long-term accuracy of occupancy-state maintenance was evaluated using five continuous sequences, each comprising 20 consecutive events. Let *N_r,j_* and ${\hat{N}}_{r,j}$ denote the ground-truth and estimated occupancy states, respectively, after the *j*-th event in the *r*-th sequence. The mean absolute occupancy error was defined as

(52) $${\mathrm{MAE}}_{\mathrm{occ}}=\frac{1}{\sum_{r=1}^{R}L_{r}}\overset{R}{\underset{r=1}{\sum }}\overset{L_{r}}{\underset{j=1}{\sum }}\left|{\hat{N}}_{r,j}-N_{r,j}\right|,$$

where *R* = 5 denotes the number of sequences and *L_r_* = 20 denotes the number of events in each sequence.

The accumulation of counter errors was also evaluated using the mean final absolute error:

(53) $$D_{\mathrm{final}}=\frac{1}{R}\overset{R}{\underset{r=1}{\sum }}\left|{\hat{N}}_{r,L_{r}}-N_{r,L_{r}}\right|.$$

The metric *D_final_* quantified the average difference between the estimated and ground-truth occupancy states at the end of a complete sequence. In addition, the ground-truth and estimated occupancy trajectories after successive events were presented for each sequence. The classification results for both people counts and the five event types were also presented as confusion matrices.

## 3. Results

The results are first presented at the individual-event level, followed by an evaluation of their effect on the long-term occupancy state. All individual-event results were calculated using the held-out test session comprising 110 events, which were not used for model training or hyperparameter selection. Counter-error accumulation was evaluated independently using five continuous sequences, each comprising 20 events.

The evaluation included the complete hybrid method, baseline methods, and ablation variants. The effects of scenario type, number of participants, and confidence threshold on counting performance and the accuracy of the maintained occupancy state were also analyzed.

### 3.1. Overall Event-Level Performance

The first experiment independently evaluated the two six-class counting heads and the five-class event-type head. *Accuracy* and *macro-F1* were calculated for each head, while the mean absolute error was additionally determined for the entry and exit counts. Joint count accuracy (*JCA*) was adopted as the most stringent criterion because it considers an event correct only when both the entry and exit counts are estimated correctly. Detailed results are presented in Table 3.

The counting heads achieved an accuracy of 95.5% for entries and 94.5% for exits, while the five-class event-type head achieved an accuracy of 97.3%. Both counts were simultaneously estimated correctly for 102 of the 110 events, corresponding to a *JCA* of 92.7%. The *MAE* of the signed occupancy change was 0.06 person per event. This value reflected the partial cancelation of errors in *n_in_* − *n_out_* for some mixed events.

**Figure 4:**
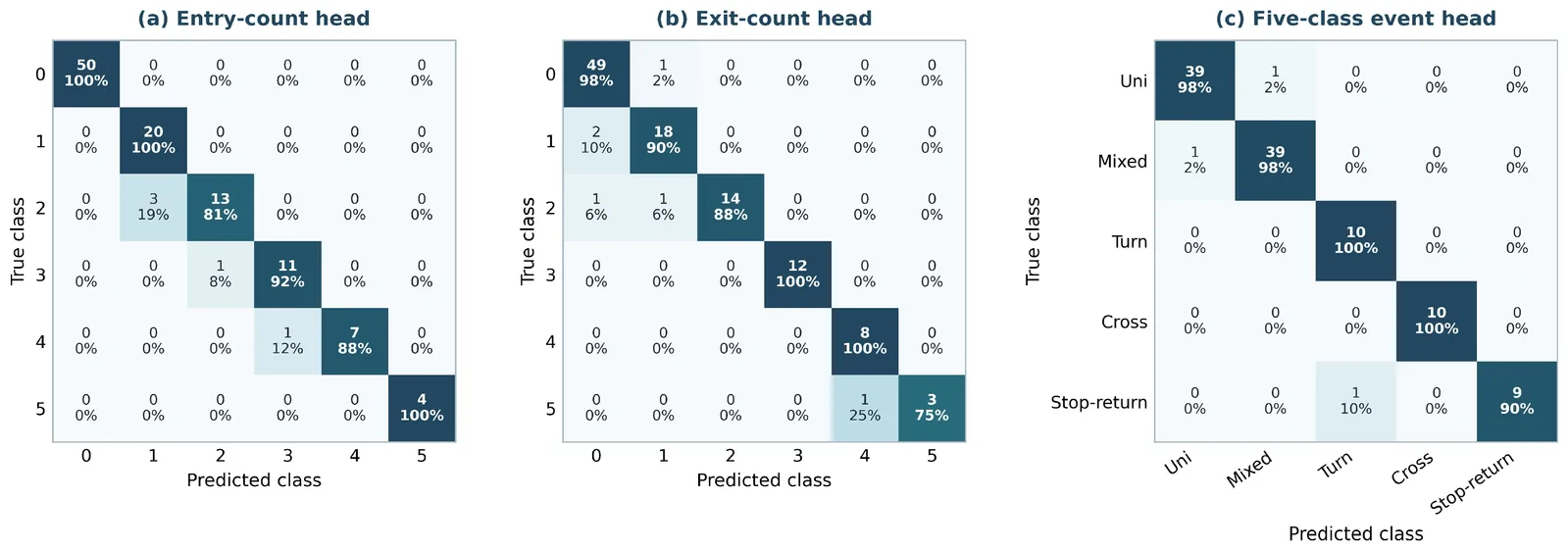
Confusion matrices for the entry-count, exit-count, and five-class event-type heads.

Errors between adjacent count classes dominated the confusion matrices, particularly for classes representing three to five people. For the event-type head, one error occurred between a reversal and a stop followed by a return toward the doorway. Both events produce zero occupancy change and differ primarily in the time spent within the entrance zone. The confusion matrices are presented in Figure 4.

### 3.2. Performance by Scenario and Group Size

In the second experiment, the results were separated into unidirectional events, mixed events, and events that did not change the occupancy state. This division made it possible to determine whether the aggregated result concealed a reduction in performance for simultaneous movement in opposite directions. The results are presented in Table 4.

The highest performance was obtained for unidirectional movement, for which the *JCA* reached 97.5%. In this case, the clear displacement of radar activity was consistent with both the features analyzed by the model and the geometric verification. The decrease in *JCA* to 85.0% for mixed events resulted from the temporary merging of reflections from multiple individuals and the need to recover the two counts independently. For events with no occupancy change, *JCA* is not the only relevant metric; maintaining *MAE_ΔN_* close to zero and preventing false counter updates are particularly important.

For the 80 test events involving actual doorway crossings, the results were additionally grouped according to the total number of participants. The numbers of events for each group size followed directly from the test-session design: eight single-person events and 12, 16, 20, and 24 events involving two, three, four, and five people, respectively. The results are presented in Table 5.

**Figure 5:**
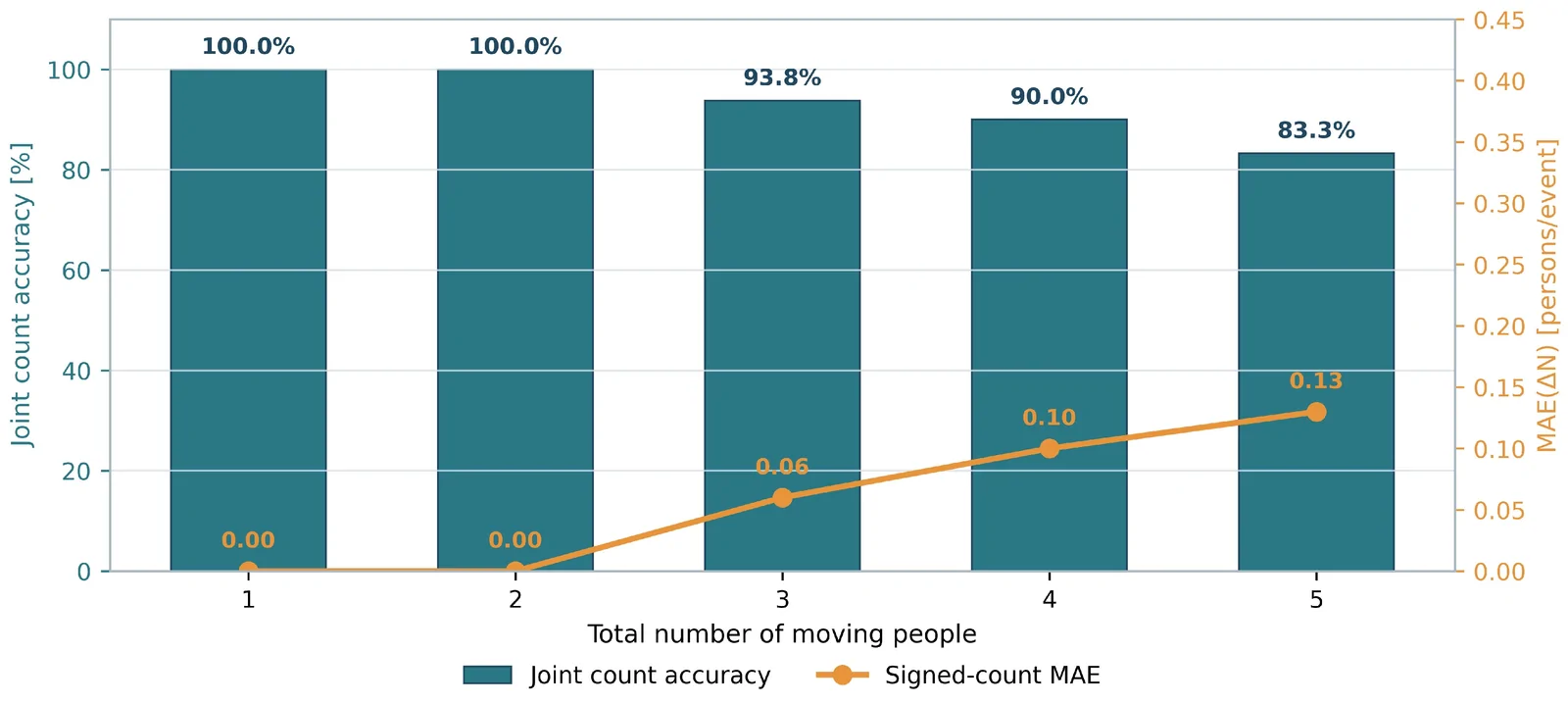
Joint count accuracy and signed-count MAE versus group size.

Performance decreased monotonically as the number of people increased. The *JCA* was 100% for one and two people and subsequently decreased to 93.8%, 90.0%, and 83.3% for three, four, and five participants, respectively. At the same time, *MAE_ΔN_* increased from 0.00 to 0.13 persons per event. These results are illustrated in the Figure 5. 

### 3.3. Comparison with Baseline Methods

The benefit of using an artificial intelligence model was assessed by comparing three variants: geometry-only rules without learning, conventional processing based on Density-Based Spatial Clustering of Applications with Noise (DBSCAN) and tracking, and the complete hybrid method. All thresholds used by the baseline methods were selected exclusively using the validation set. For the long-term comparison, each variant was applied to the same five continuous sequences. The results are presented in Table 6.

The complete method increased *JCA* by 37.2 percentage points relative to the geometry-only rules and by 24.5 percentage points relative to DBSCAN combined with tracking. From a practical perspective, the reduction *MAE_occ_* in was also important. Individual errors in conventional trajectory association accumulate in the counter, whereas geometric verification and the rejection of uncertain events in the hybrid method reduce the number of incorrect updates.

### 3.4. Ablation Study

The ablation study determined which components of the architecture were responsible for the performance improvement. Each variant was trained from scratch using the same session-based dataset split and optimizer settings. The evaluated components were radial velocity, BiGRU-based temporal modeling, the attention mechanism, the auxiliary five-class head, geometric verification, and the confidence criterion. The results are presented in Table 7.

The largest performance degradation occurred after removing the BiGRU, supporting the main hypothesis of this study: information describing the complete temporal evolution of an event is required to distinguish an actual passage from a reversal or the temporary merging of reflections. The attention mechanism and radial velocity provided smaller but measurable improvements. Removing geometric verification or the confidence criterion did not change the raw *JCA* of the network heads but increased the occupancy-state error because more incorrect updates were applied.

### 3.5. Confidence–Coverage Analysis

The confidence threshold was evaluated using more than the accuracy of the accepted events alone. A threshold that is too low increases the number of incorrect updates, whereas an excessively high threshold also rejects correctly recognized passages and may cause a persistent difference between the ground-truth and estimated occupancy states. Therefore, coverage, selective accuracy, and *MAE_occ_* were determined simultaneously for successive threshold values. The results are presented in Table 8.

**Figure 6:**
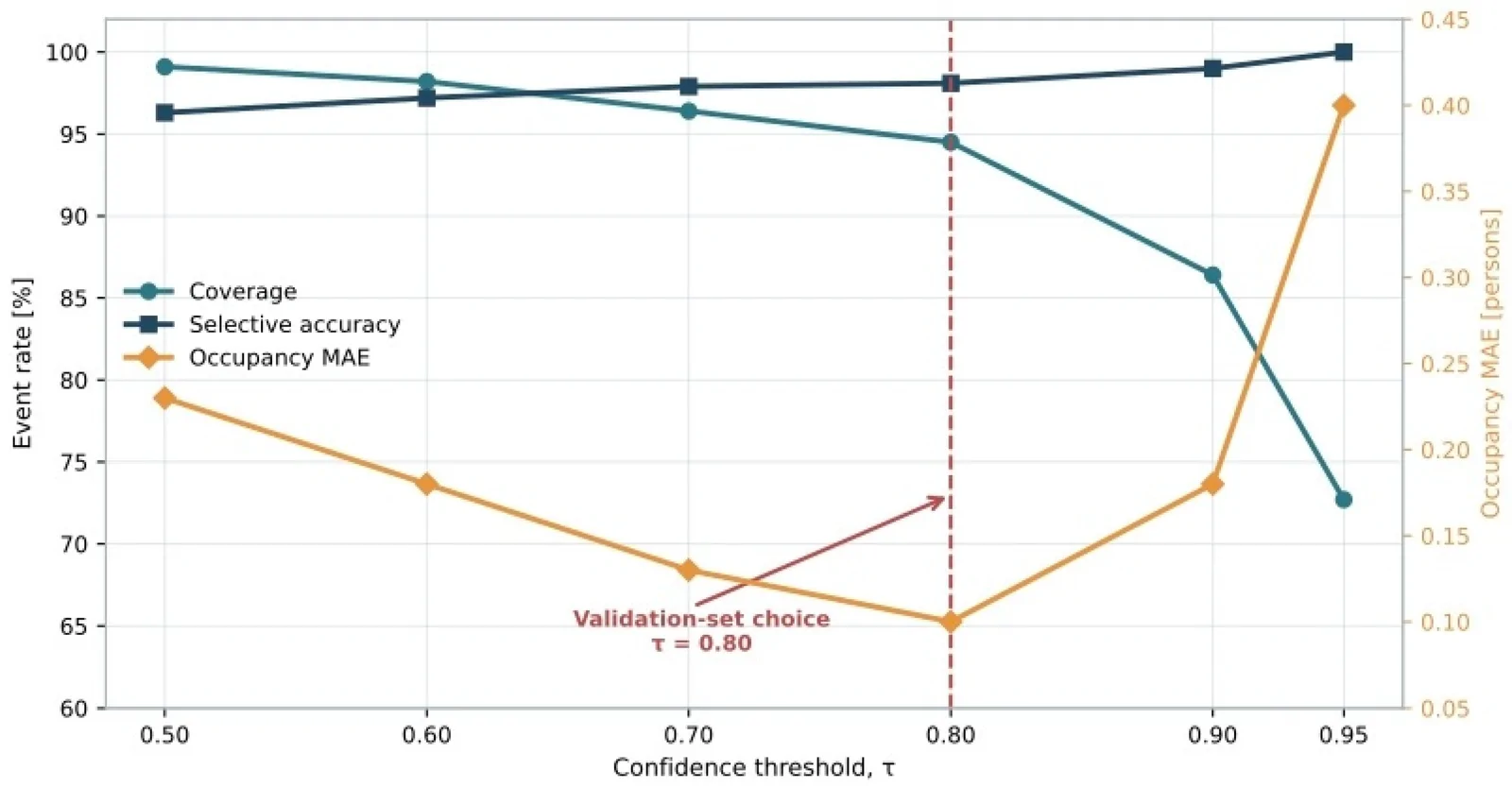
Selective accuracy–coverage characteristic and occupancy error versus confidence threshold.

The most favorable trade-off was obtained for *τ* = 0,80, which was selected exclusively using the validation set. Further increasing the threshold improved selective accuracy but reduced coverage to such an extent that occupancy error increased again. At *τ* = 0,80, the method achieved a coverage of 94.5%, a selective accuracy of 98.1%, and the lowest *MAE_occ_*, equal to 0.10 person. These relationships are also illustrated in the Figure 6.

### 3.6. Long-Term Occupancy Evaluation

The most important system-level evaluation involved five continuous sequences, each comprising 20 consecutive events and beginning from a known occupancy state. After each event, the estimated state was compared with the reference state, after which *MAE_occ_* and the absolute final error were calculated. A rejected event does not constitute an incorrect update but may produce a subsequent discrepancy in the occupancy state; therefore, these two effects were reported separately. The results are presented in Table 9.

**Figure 7:**
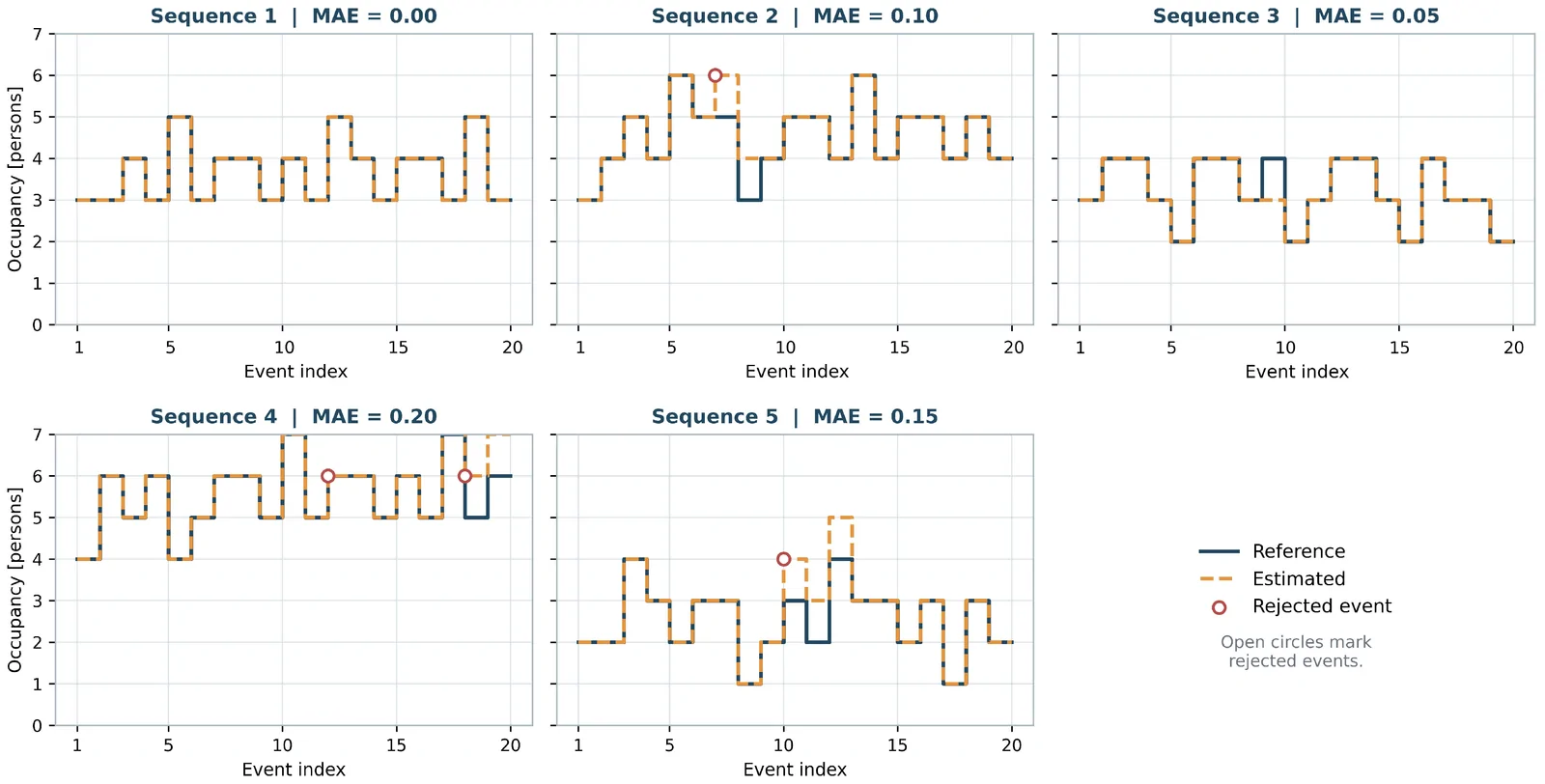
Reference and estimated occupancy trajectories for the five continuous sequences.

The mean *MAE_occ_* was 0.10 person, while the mean absolute final error was 0.20 person per sequence. In the first sequence, the occupancy state was reconstructed without error, whereas the highest *MAE_occ_*, equal to 0.20 person, was recorded for the fourth sequence. The reference and estimated occupancy trajectories show that an individual error could either be compensated for by a subsequent event or persist until the end of the measurement sequence. These results are illustrated in Figure 7.

### 3.7. Cross-Environment Generalization Evaluation

To evaluate cross-environment generalization, the model trained and selected exclusively using data collected in the original laboratory was tested using an independent dataset recorded in a second laboratory. No data from the second environment were used for model training, validation, hyperparameter selection, or decision-threshold determination. The model was applied without retraining or fine-tuning, and all preprocessing parameters, event-segmentation settings, geometric-consistency rules, and the confidence threshold were kept unchanged.

The second laboratory differed from the original environment in terms of room geometry, furnishings, distribution of reflecting objects, and doorway-zone configuration. The independent dataset comprised 100 complete doorway events, with 20 events representing each of the five event categories considered in the original evaluation.

**Figure 8:**
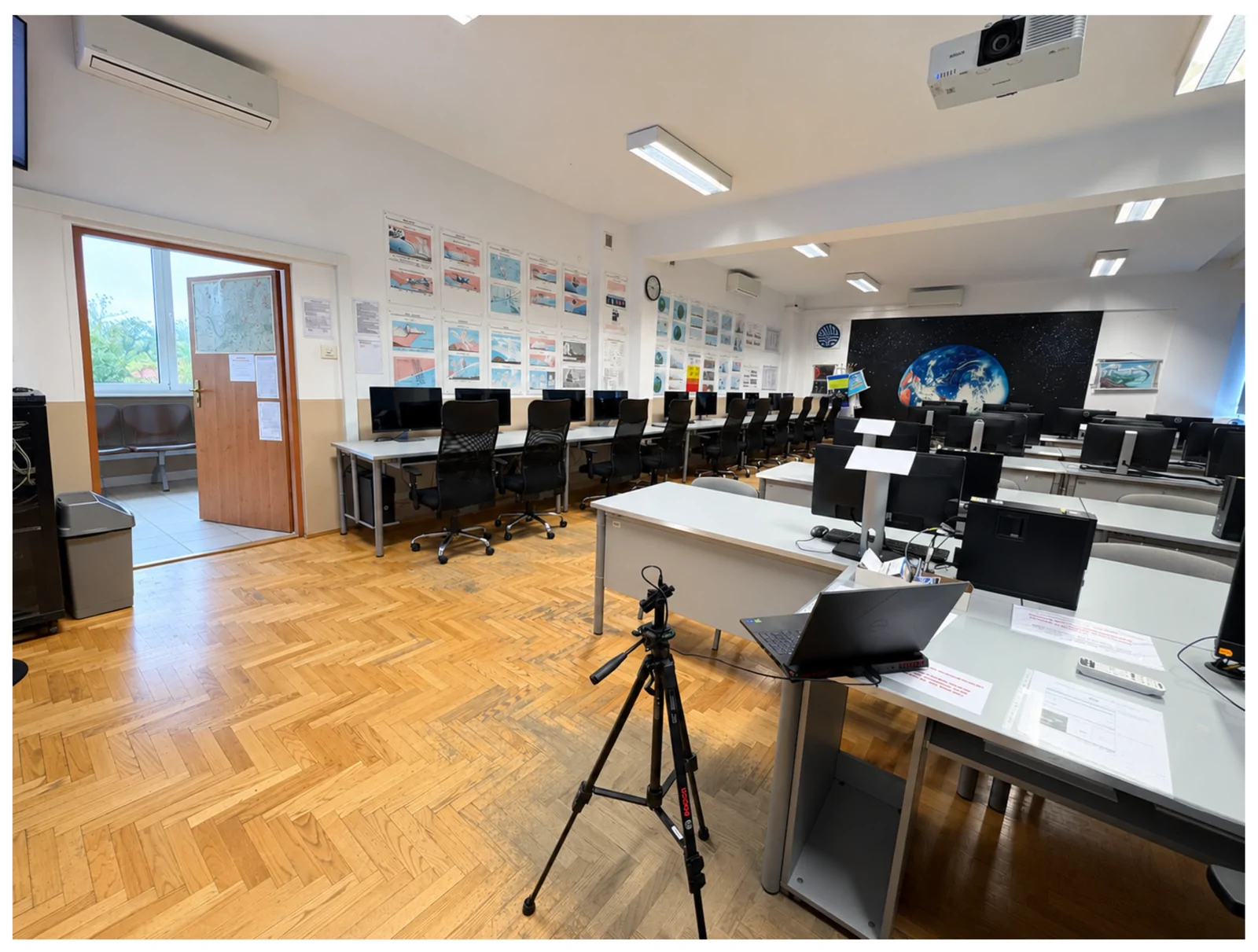
Independent laboratory used for the cross-environment generalization evaluation.

The second laboratory measured 12 × 7 m, and the monitored doorway was approximately 1.2 m wide. The measurement environment and radar placement are shown in Figure 8.

The results obtained in the original and independent laboratory environments are compared in Table 10. The joint entry–exit count accuracy decreased from 92.7% in the original held-out test session to 91.0% in the independent laboratory, corresponding to a reduction of 1.7 percentage points. This limited decrease indicates that the model retained its ability to estimate entry and exit counts despite changes in the measurement environment. Importantly, this result was obtained without using any data from the second room for training, validation, hyperparameter selection, or model adaptation. The event-level accuracies reported in Table 10 were calculated for all 100 events using the outputs of the three classification heads before confidence-based rejection.

Nevertheless, the experiment involved only one additional environment and should therefore be interpreted as preliminary evidence of cross-environment generalization rather than comprehensive validation across different rooms, doorway widths, and radar mounting configurations.

## 4. Discussion, Limitations and Future Work

The results support the formulation of bidirectional people counting as the analysis of complete events rather than the classification of individual frames or the continuous tracking of individual trajectories. The complete method achieved accuracies of 95.5% and 94.5% for the entry and exit counts, respectively. Both counts were simultaneously estimated correctly for 102 of the 110 test events, corresponding to a joint entry–exit count accuracy (JCA) of 92.7%. The mean absolute error of the signed occupancy change was 0.06 persons per event. The difference between the accuracies of the individual counting heads and the JCA demonstrates that evaluating entry and exit counts separately may lead to an overly optimistic interpretation of performance. Maintaining an accurate occupancy state requires both counts to be estimated correctly within the same event.

Comparison with the baseline methods showed that geometric information alone was insufficient to interpret complex events. The complete method increased JCA by 37.2 percentage points relative to the geometry-only rules and by 24.5 percentage points relative to the approach based on DBSCAN clustering and tracking. At the same time, the occupancy-state MAE decreased from 1.10 and 0.78 persons, respectively, to 0.10 person. These results confirm that the number of radar points or temporarily identified clusters is not a reliable proxy for the number of people, particularly when reflections from several participants periodically merge into a single extended object.

The advantage of the event-based approach can be explained by the different information required by the two processing strategies. A conventional tracker depends on the continuity of separate detections and on correct frame-to-frame data association. When reflections from people moving close to one another temporarily merge, the number of detected clusters may decrease, existing tracks may be lost, and the subsequent separation of reflections may produce ambiguous track assignments. Simultaneous movement in opposite directions creates an additional difficulty because detections associated with different directions may overlap spatially near the doorway threshold. In contrast, the proposed method does not require point-to-point correspondence, continuous individual tracks, or persistent person identities. It uses the spatial distribution of the complete point cloud, radial velocity, and their temporal evolution before, during, and after the period of reduced separability.

Mechanistically, the spatial coordinates describe changes in the geometry and location of radar activity, including its redistribution between the entrance and inner zones. Radial velocity provides information about the direction and dynamics of movement relative to the radar. The PointNet encoder transforms each point cloud into a frame-level representation without assigning detections to individual people. The BiGRU integrates the evolution of these representations across the complete event, while the attention mechanism allows the model to assign greater importance to temporal segments associated with doorway crossing, direction changes, temporary merging or separation of reflections, and departure from the monitored region. Consequently, information available before and after a temporary loss of separability can contribute to the final decision.

The highest accuracy was obtained for unidirectional movement, for which the JCA reached 97.5%. For simultaneous movement in opposite directions, this value decreased to 85.0%. Mixed events therefore remain the most challenging scenario because the model must independently recover two counts from partially overlapping reflections. The deterioration in performance with increasing group size had a similar cause. The JCA was 100% for events involving one or two people but decreased to 83.3% for five people. This indicates that the primary limitation is not recognition of the general direction of movement but separation of the contributions of individual people under mutual occlusion and with a limited number of radar-generated points.

The five-class event-type head achieved an accuracy of 97.3%, demonstrating the ability to distinguish actual passages and mixed events from behaviors that did not change the occupancy state. This is particularly important for reversals, lateral passages, and stops followed by a return toward the doorway because their initial segments may resemble an actual entry or exit. Auxiliary event-type classification reduced the risk of interpreting short-term activity near the doorway as a completed threshold crossing. This was confirmed by the ablation study, in which removing the auxiliary head reduced JCA from 92.7% to 88.2% and increased the long-term occupancy-state error.

The largest deterioration in the ablation study occurred after removing the BiGRU network. The JCA decreased to 80.0%, while the occupancy-state MAE increased from 0.10 to 0.42 person. This indicates that a single-frame representation does not contain sufficient information to reliably recognize the complete course of an event. Radial velocity and the attention mechanism were also important. Removing radial velocity reduced JCA to 88.2%, whereas removing the attention mechanism reduced it to 89.1%. Radial velocity supplemented geometric information with movement dynamics, while the attention mechanism allowed greater importance to be assigned to sequence segments associated with doorway crossing, direction changes, or departure from the monitored area.

The interpretability analysis presented in this study remains limited to a mechanistic interpretation of the input features and network components supported by the ablation results. Intermediate representations, event-specific attention weights, and frame-level model responses were not archived during the original experiments. Consequently, these quantities could not be reliably reconstructed for the events included in the reported evaluation without retraining the model and obtaining a different model realization. Rather than presenting newly generated visualizations as results of the original experiment, the revised manuscript provides a more detailed explanation of how spatial features, radial velocity, and temporal changes contribute to the final decisions. Future work should include the systematic recording and visualization of intermediate representations, attention distributions, and key-frame responses for representative mixed events and cases involving temporary merging of reflections.

Geometric-consistency verification and the confidence criterion had the greatest effect on long-term occupancy-state accuracy. Removing them did not change the raw JCA of the neural-network outputs but increased the occupancy-state MAE to 0.22 and 0.27 person, respectively. This demonstrates that individual-event classification accuracy does not fully describe the utility of a counting system. Even a small number of incorrect decisions may accumulate over successive updates; therefore, the ability to reject an ambiguous event is an important component of the method. The most favorable trade-off was obtained using a confidence threshold of 0.80, for which coverage reached 94.5%, selective accuracy reached 98.1%, and the occupancy-state MAE was 0.10 person. Further increasing the threshold improved the accuracy of accepted decisions but rejected too many actual passages, causing the counter error to increase again.

Evaluation using five continuous sequences confirmed that the method could maintain the occupancy state without continuously detecting the people present in the room. Across 100 consecutive events, the occupancy-state MAE was 0.10 person, and the mean absolute final error was 0.20 person per sequence. This result is relevant to applications such as heating, ventilation, and air-conditioning control, which require information about current occupancy rather than the continuous localization of every occupant. However, the method maintains the occupancy state on the basis of accepted entry and exit events and does not directly detect stationary people already present in the room.

The cross-environment experiment provided additional evidence regarding the transferability of the proposed method. When the model trained exclusively using data from the original laboratory was applied to 100 events recorded in the second laboratory, the joint entry–exit count accuracy decreased from 92.7% to 91.0%. Entry-count, exit-count, and event-type accuracies reached 94.0%, 92.0%, and 96.0%, respectively, while the mean absolute error of the signed occupancy change increased only from 0.06 to 0.07 person per event. These results were obtained without retraining, fine-tuning, parameter adaptation, or modification of the decision thresholds. The limited performance reduction suggests that the model captured spatiotemporal characteristics of complete doorway events that were not restricted to the geometry of the original laboratory. Nevertheless, because the evaluation involved only one additional environment, this result should be regarded as preliminary evidence of cross-environment generalization.

Direct comparison of the obtained values with the results reported in studies [15,16,17,18,19,20,21,22,23,24,25] requires caution. The cited methods use different radar frequencies, signal representations, class definitions, numbers of participants, measurement geometries, and data-partitioning procedures. Some evaluate trajectory-tracking accuracy, whereas others evaluate scene-count classification or movement-direction recognition. In contrast, the results presented in this study concern the simultaneous estimation of entry and exit counts within a complete event and the resulting occupancy-state updates. The performance gains should therefore be assessed primarily relative to the baseline methods evaluated using the same test dataset.

The first limitation concerns the number and diversity of the measurement environments. Although the model was additionally evaluated in an independent laboratory without retraining or parameter adaptation, the study included only two rooms and two doorway configurations. The cross-environment experiment therefore provides preliminary evidence of generalization rather than comprehensive validation across different buildings and operating conditions. Further experiments are required to determine the robustness of the method to wider variations in room geometry, doorway width, equipment arrangement, radar distance, mounting height, mounting angle, and measurement-region configuration.

The second limitation is the dataset size. The model was developed using 330 training events, and the primary evaluation was conducted using a single held-out test session comprising 110 events. This partitioning enabled an independent session-level evaluation, but the results for less frequent configurations, particularly groups of four and five people, are subject to greater uncertainty. The independent laboratory dataset contained an additional 100 events, but these measurements were intended specifically for cross-environment evaluation and did not increase the diversity of the training data. Future studies should increase the number of sessions, participants, and repetitions and should evaluate several independent session-based splits or apply leave-one-session-out validation. Reporting confidence intervals for the principal metrics would also be appropriate.

The dataset comprised five predefined event categories and did not cover all behaviors that may occur at a real doorway. The study did not consider, among other cases, people carrying large objects, strollers or carts, children and adults with substantially different body sizes, prolonged presence within the doorway zone, or consecutive groups whose radar activity did not disappear between passages. In such cases, the event-segmentation mechanism may combine several passages into a single long sequence. Extending the dataset with events outside the known class set should be accompanied by a method for detecting out-of-distribution cases.

The adopted maximum sequence length and number of points also constitute limitations. Events were represented using no more than 80 frames and 64 points per frame. Farthest point sampling preserves the spatial extent of the point cloud but may remove weaker reflections associated with some participants. Similarly, uniform sampling of events lasting longer than 8 s may reduce the visibility of brief motion phases relevant to recognizing a doorway crossing. Future work should evaluate the effects of temporal resolution and point count and consider models capable of processing variable-length sequences without reducing them to a fixed size.

The use of a bidirectional GRU network means that a decision can be made only after an event has ended. This is consistent with the adopted counter-update procedure but prevents the final occupancy change from being determined while a passage is still in progress. Moreover, inference time, memory consumption, and model operation on a target embedded platform were not evaluated separately. Before the system can be used to control building equipment, its total latency must be determined, including event-start and event-end detection, tensor preparation, model inference, geometric-consistency verification, and occupancy-state updating.

The method uses point clouds generated by the radar’s OOB software. This facilitates implementation and eliminates the need to process raw ADC samples, but it also makes the input quality dependent on the detector configuration and the algorithms implemented in the device. Reflections from partially occluded individuals may be discarded before the data are passed to the model. Generalization to other radar devices or configurations will therefore require verification of data-format compatibility, detection characteristics, and the distribution of point counts.

The long-term evaluation comprised a total of 100 events, which made it possible to observe error accumulation and compensation but did not represent multi-day system operation. Because the counter is updated recursively, a single missed entry or exit may maintain a discrepancy between the ground-truth and estimated occupancy states over many subsequent events. Rejecting events protects the system from updates based on uncertain decisions but may also cause temporary occupancy errors. A practical deployment may therefore require a mechanism for periodic correction or reinitialization of the occupancy state, for example, following the controlled emptying of the room or using an additional independent information source.

Future work should include measurements in additional rooms with more diverse doorway widths and radar mounting configurations, larger numbers of participants and scenarios, evaluation of cross-subject generalization, and longer continuous sequences. It will also be important to investigate causal models that make decisions using only the observed portion of an event, adaptive confidence criteria, methods for detecting out-of-distribution events, and procedures for automatically correcting the occupancy state. Systematic recording and visualization of intermediate representations, attention distributions, and key-frame responses should also be included to provide a more direct interpretation of model decisions. These studies will help determine the extent to which the proposed method can be transferred from controlled laboratory environments to long-term operation in real buildings.

## 5. Conclusions

This study developed an event-based method for bidirectional people counting and room occupancy estimation using OOB point clouds generated by a single 77 GHz MIMO FMCW radar. Rather than continuously tracking individual people, the proposed approach treats a complete doorway passage as a single event. The PointNet encoder represents the spatial distribution of radar points in individual frames, the BiGRU models the temporal evolution of these representations before, during, and after temporary merging of reflections, and the attention mechanism assigns greater importance to the most informative phases of the event. Separate output heads estimate the numbers of entering and exiting people, while geometric-consistency verification and the confidence criterion reduce the risk of incorrect occupancy-state updates.

In the original held-out test session, the complete method achieved a joint entry–exit count accuracy of 92.7% and a mean absolute error of 0.06 persons per event for the signed occupancy change. Across five continuous sequences comprising 100 events, the mean occupancy-state MAE was 0.10 person. The comparative and ablation studies confirmed the importance of temporal modeling and demonstrated the advantage of the proposed hybrid method over geometry-only rules and an approach based on DBSCAN clustering and tracking.

In the independent laboratory, the model achieved a joint entry–exit count accuracy of 91.0% and a mean absolute error of 0.07 persons per event without retraining, fine-tuning, or parameter adaptation. This limited performance reduction provides preliminary evidence that the learned event representation can be transferred between different measurement environments. Nevertheless, further evaluation involving additional rooms, doorway geometries, radar mounting configurations, participants, and longer continuous measurements is required before the method can be considered suitable for general real-world deployment.

## Figures and Tables

**Table 1:** Radar parameters and FMCW waveform configuration used in the experiments.

Parameter	Value
Platform frequency range	76–81 GHz
Profile start frequency	77 GHz
Equivalent isotropically radiated power (EIRP)	19 dBm
Number of active transmit antennas	3
Number of active receive antennas	4
Azimuth field of view	120°
Elevation field of view	40°
Chirp frequency slope	70 MHz/µs
Chirp ramp time	57.14 µs
Idle time between chirps	267 µs
ADC sampling start delay	7 µs
Number of ADC samples per chirp	256
Sampling rate	5.209 MS/s
Total chirp sweep bandwidth	4.00 GHz
Effective sampled bandwidth	3.44 GHz
Range resolution	0.044 m
Number of MIMO cycles per frame	16
Number of chirps per frame	48
Frame period	100 ms
Frame rate	10 Hz
Maximum unambiguous range	9.02 m
Configured range interval	0–8.92 m
Maximum unambiguous radial velocity	±1.00 m/s
Velocity resolution	0.13 m/s
Receiver gain	30 dB

**Table 2:** Composition of the event-based radar dataset.

Event Type	Number of Configurations	Repetitions per Configuration	Total Number of Events
Unidirectional movement of one to five people between the entrance zone and the laboratory interior	10	20	200
Simultaneous movement of people in opposite directions	10	20	200
Reversal within the entrance zone	1	50	50
Lateral passage through the entrance zone	1	50	50
Stop within the entrance zone followed by a return toward the doorway	1	50	50
Total	23	-	550

**Table 3:** Event-level performance on the held-out test session.

Evaluated Output	Number of Events	Accuracy (%)	Macro-F1	MAE (Persons/Event)
Entry count *n_in_*	110	95.5 (105/110)	0.941	0.05
Exit count *n_out_*	110	94.5 (104/110)	0.932	0.06
Event type (5 classes)	110	97.3 (107/110)	0.970	—
Joint entry–exit count accuracy (JCA)	110	92.7 (102/110)	—	—
Signed occupancy change ΔN	110	—	—	0.06

**Table 4:** Performance by scenario on the held-out test session.

Scenario Type	Number of Events	JCA [%]	MAE_ΔN_ (Persons/Event)	Event-Type Macro-F1
Unidirectional movement	40	97.5 (39/40)	0.03	0.98
Simultaneous movement in both directions	40	85.0 (34/40)	0.13	0.97
No occupancy change	30	96.7 (29/30)	0.03	0.95
Overall	110	92.7 (102/110)	0.06	0.97

**Table 5:** Count performance versus the total number of moving people.

Total Number of People	Number of Events	JCA [%]	MAE_ΔN_ [Persons/Event]
1	8	100.0	0.00
2	12	100.0	0.00
3	16	93.8	0.06
4	20	90.0	0.10
5	24	83.3	0.13

**Table 6:** Comparison with non-neural baseline methods.

Method	JCA [%]	MAE_ΔN_	False Updates for No-Change Events [%]	*MAE_occ_* [Persons]
Geometry-only rules	55.5	0.64	20.0	1.10
DBSCAN + tracking	68.2	0.45	13.3	0.78
Complete hybrid method	92.7	0.06	3.3	0.10

**Table 7:** Ablation results for the proposed pipeline.

Variant	JCA [%]	*MAE_ΔN_*	Selective Accuracy [%]	Coverage [%]	*MAE_occ_* [Persons]
Complete method	92.7	0.06	98.1	94.5	0.10
Without radial velocity	88.2	0.12	96.0	93.6	0.20
Without BiGRU	80.0	0.25	92.0	87.3	0.42
Without the attention mechanism	89.1	0.11	96.4	92.7	0.18
Without the auxiliary five-class head	88.2	0.14	95.0	91.8	0.24
Without geometric verification	92.7	0.06	96.2	96.4	0.22

**Table 8:** Confidence–coverage trade-off.

Confidence Threshold τ	Coverage [%]	Selective Accuracy [%]	*MAE_occ_* [Persons]
0.50	99.1	96.3	0.23
0.60	98.2	97.2	0.18
0.70	96.4	97.9	0.13
0.80 (selected using the validation set)	94.5	98.1	0.10
0.90	86.4	99.0	0.18
0.95	72.7	100.0	0.40

**Table 9:** Long-term occupancy results for five continuous sequences.

Sequence	Number of Events	Accepted Events	Rejected Events	Incorrect Updates	*MAE_occ_* [Persons]	Final Error [Persons]
1	20	20	0	0	0.00	0
2	20	19	1	0	0.10	0
3	20	20	0	1	0.05	0
4	20	18	2	1	0.20	1
5	20	19	1	0	0.15	0
Mean/total	20/100	19.2/96	0.8/4	0.4/2	0.10	0.20

**Table 10:** Comparison of event-level performance in the original and independent laboratory environments.

Evaluated Output	Original Laboratory	Independent Laboratory	Change
Entry-count accuracy	95.5%	94.0%	−1.5 pp
Exit-count accuracy	94.5%	92.0%	−2.5 pp
Event-type accuracy	97.3%	96.0%	−1.3 pp
Joint entry–exit count accuracy (JCA)	92.7%	91.0%	−1.7 pp
MAE of signed occupancy change ΔN	0.06	0.07	+0.01

## Data Availability

The data supporting the findings of this study are available from the corresponding author upon reasonable request.

## Abbreviations

The following abbreviations are used in this manuscript:

ADC	analog-to-digital converter
AI	artificial intelligence
BiGRU	bidirectional gated recurrent unit
BN	batch normalization
CFAR	constant false-alarm rate
CNN	convolutional neural network
CVD	cadence–velocity diagram
DBSCAN	density-based spatial clustering of applications with noise
EIRP	equivalent isotropically radiated power
FMCW	frequency-modulated continuous-wave
FN	false negative
FP	false positive
FPS	farthest point sampling
GRU	gated recurrent unit
HPA	high-performance azimuth
HVAC	heating, ventilation, and air conditioning
IR-UWB	impulse-radio ultra-wideband
JCA	joint count accuracy
LSTM	long short-term memory
MAE	mean absolute error
MCMCDA	Markov chain Monte Carlo data association
MFSNet	multiscale feature space fusion network
MIMO	multiple-input multiple-output
OOB	out-of-box
PIR	passive infrared
ReLU	rectified linear unit
ResNet-50	50-layer residual network
RGB	red–green–blue
RPCNet	radar-based people-counting network
SDK	software development kit
SNN	spiking neural network
SNR	signal-to-noise ratio
TLV	type–length–value
TP	true positive
UART	universal asynchronous receiver-transmitter
USB	universal serial bus
VGG16	visual geometry group 16-layer network
